# A first look at Android applications in Google Play related to COVID-19

**DOI:** 10.1007/s10664-021-09943-x

**Published:** 2021-04-21

**Authors:** Jordan Samhi, Kevin Allix, Tegawendé F. Bissyandé, Jacques Klein

**Affiliations:** University of Luxembourg, SnT, 6 Rue Richard Coudenhove-Kalergi, 1359 Luxembourg, Luxembourg

**Keywords:** COVID-19, Coronavirus, Android apps, Statistics

## Abstract

Due to the convenience of access-on-demand to information and business solutions, mobile apps have become an important asset in the digital world. In the context of the COVID-19 pandemic, app developers have joined the response effort in various ways by releasing apps that target different user bases (e.g., all citizens or journalists), offer different services (e.g., location tracking or diagnostic-aid), provide generic or specialized information, etc. While many apps have raised some concerns by spreading misinformation or even malware, the literature does not yet provide a clear landscape of the different apps that were developed. In this study, we focus on the Android ecosystem and investigate Covid-related Android apps. In a best-effort scenario, we attempt to systematically identify all relevant apps and study their characteristics with the objective to provide a first taxonomy of Covid-related apps, broadening the relevance beyond the implementation of contact tracing. Overall, our study yields a number of empirical insights that contribute to enlarge the knowledge on Covid-related apps: (1) Developer communities contributed rapidly to the COVID-19, with dedicated apps released as early as January 2020; (2) Covid-related apps deliver digital tools to users (e.g., health diaries), serve to broadcast information to users (e.g., spread statistics), and collect data from users (e.g., for tracing); (3) Covid-related apps are less complex than standard apps; (4) they generally do not seem to leak sensitive data; (5) in the majority of cases, Covid-related apps are released by entities with past experience on the market, mostly official government entities or public health organizations.

## Introduction

The outbreak of the COVID-19 pandemic in late December 2019 has quickly and severely impacted countries worldwide, becoming one of the most important health crisis of the 21^*st*^ century (Spinelli and Pellino 2020; Remuzzi and Remuzzi 2020). The infectious agent, a *coronavirus*, identified as responsible for this disease is notoriously difficult to pin down: while it leaves many infected people without symptoms, it can lead to a common cold for some and even severe respiratory disorders to others (Clerkin et al. 2020). So far, the COVID-19 has brought about a human tragedy, with hundreds of thousands of lives lost, as well as an economic downturn due to the lockdown of over three billion people (half of humanity) (Mahase 2020; Dudel et al. 2020).

The scale of COVID-19 effects has urged stakeholders at different levels (government, local authorities, private companies, academia, NGOs, and citizens) to plan and implement measures for addressing the virus spread. In particular, while pharmaceutical research is embarked on a long journey to develop a vaccine, non-pharmaceutical innovations are sought to contribute to responding to the outbreak. Chief among the technologies that are employed, Information and Communication Technology has been widely leveraged in various capacities across all regions and targeting broadly all levels of society. For example, news and sensitization messages have been viral thanks to the use of internet-based services such as social networks. Given the widespread use of handheld devices such as smartphones, users are keen to install applications (often referred to as *apps* in the mobile realm) that have specific purposes for entertainment, business, productivity, news, and social networking. Following the outbreak of the pandemic, authorities, non-governmental organizations, and independent developers have engaged in an app development race to provide readily-available digital tools to the modern citizen.

We focus in this paper on the case of the Android ecosystem. With the largest market share on mobiles (86% in 2020 (IDC 2020)), Android constitutes a prime choice for developers and users alike. Initial reporting on COVID-19 related apps are focused on the problems that such apps raise: (1) He et al. (2020) have already explored the case of coronavirus-themed Android malware; (2) Google, the maintainer of the Google Play (i.e., the official Android app market) has decided to crack down on COVID-19 apps to combat misinformation, sometimes with an excessive zeal (i.e., legitimate apps can be temporarily banned just for sharing COVID-19 information (Carman 2020; Google 2020a)).

Our study is of a broader and more generic dimension. It is about **characterizing the applications that are related to the COVID-19 outbreak**: What are they for? Who developed them? To what extent can they be considered dangerous? These are some of the questions that we undertake to investigate. To that end, we have considered 184563 apps released in the time window of July 2019-May 2020 and collected in the AndroZoo dataset (Allix et al. 2016). From this initial set of Android apps, by following well-defined heuristics, we were able to identify 44 Covid-related apps. Given the limitations of the crawling of AndroZoo, we also considered other online resources such as the GitHub search engine, specialized COVID-19 technology blogs, etc., and we finally raise the number of collected Covid-related apps to 92. We extract different features from these apps and provide summary statistics on their characterization.

This paper presents our analyses exploring the use of permissions and libraries, the presence of leaks, the malicious status, the code size and complexity, the authorship, and the described purposes. Mainly, we establish that most of the apps are made for informing people, monitoring their health, and tracing users with the goal of preventing the spread of the virus. In addition, we note that Covid-related apps are not flagged by malware detectors. Yet, we found that some of them have been removed from Google Play. Then, after assessing the complexity of Covid-related apps and comparing them with standard apps, we found that on average, Covid-related apps are less complex, which has been shown to often be indicative of quality for apps (Jošt et al. 2013). Finally, we applied state-of-the-art security and privacy scanners in order to check for potential cryptographic API misuses and data leaks in the code.

### Messages by Audience

#### Broad public

Our empirical study builds on a dataset collected strictly based on keywords, which we later curated to have an accurate but complete view on the development of apps to address COVID-19 challenges. A main finding of our study is that our taxonomy of COVID-19 apps highlights a variety of purposes, while the current definition of COVID-19 apps on Wikipedia remains narrowly focused on “contact-tracing”. We expect that this systematic inference of knowledge from app markets will provide a broader understanding of what is a COVID-19 app to the general public. More specifically yields, our study yields a characterization of Covid-related apps for researchers, discusses qualitative analyses of Covid-related apps for developers and provides useful information about Covid-related apps exposed to end-users.

**Researchers**: The COVID-19 outbreak is a singular event in modern history that affected various domains. In the industry of software development, it is a rare occasion where a worldwide effort was put to produce apps to address the pandemic. Datasets and insights of this sudden and widespread production are valuable to the research community. Our empirical study offers a first look at Covid-related apps, which are developed in a fast-paced manner, for a variety of purposes, for different user groups, on-behalf of different stakeholders, with different business models, etc. We provide a taxonomy in this respect.
**Developers**: Development of apps is a continuous effort that can be guided with lessons learned from the successes and failures of similar apps. Our study yields a number of insights and empirical discussions on code quality and privacy issues. We even show that Google applies some strict policies to remove “undesirable” Covid-related apps from its market, providing a clear warning of what developers should pay attention to when planning to develop Covid-related apps. We further provide at the end of this study, different empirical results related to code quality and privacy. Typically, we discuss how developers can expose potential sensitive information that can be leaked during app execution.
**End-users**: Our taxonomy, by highlighting the purposes of different Covid-related apps, their processing of private information and the medium used to share information, provides a clear, informative and multi-dimensional view for users wishing to adopt Covid-related apps.

In summary, we present the following contributions:

- We present the first systematic study of Covid-related apps that explores their characteristics and compare them with other non-Covid-related apps.
- We build a taxonomy of Covid-related apps based on their described goals.
- We apply literature analysis tools on Covid-related apps and discuss their results.

All artifacts are made available online at:

https://github.com/JordanSamhi/APKCOVID

### Research Questions

The main objective of this work is to analyze and understand Covid-related apps. To do so, we empirically observe apps characteristics by extracting features that provide insights toward understanding those apps. Hence, to accomplish this objective, we plan to answer the following research questions:

- **RQ 1:** What are Covid-related apps used for?
- **RQ 2:** Do Covid-related apps have specific characteristics?
- **RQ 3:** Are Covid-related Android apps more complex than standard apps?
- **RQ 4:** To what extent were Covid-related apps removed from the official Google Play and why?
- **RQ 5:** Who are Covid-related apps’ developers?
- **RQ 6:** Do Covid-related apps have security issues?

The remainder of this paper is organized as follows. First, we present an initial Android apps dataset and some background for the reader in Section 2. Then, in Section 3, we give details about the experimental setup of our study. In Section 4, we provide the results obtained from our experiments. We discuss the threats to validity of our study in Section 5. Finally, we discuss related work in Section 6 and conclude in Section 7.

## Dataset and Background on App Analysis

### Initial Dataset

In order to perform our experiments and to exhaustively answer the research questions presented in Introduction, we need to rely on a comprehensive dataset. Consequently, we used the state-of-the-art largest dataset available, namely AndroZoo (Allix et al. 2016). At the time of writing, AndroZoo contains more than 11 million Android apps (June 2020) that have been collected from different sources, such as Google Play and other third-party providers (F-Droid, Anzhi, AppChina, etc.). As it is continuously growing, researchers still heavily rely on AndroZoo for collecting apps and experimenting on it (Ranganath and Mitra 2020; Shar et al. 2020; He et al. 2020; Xu et al. 2020).

**Fig. 1:**
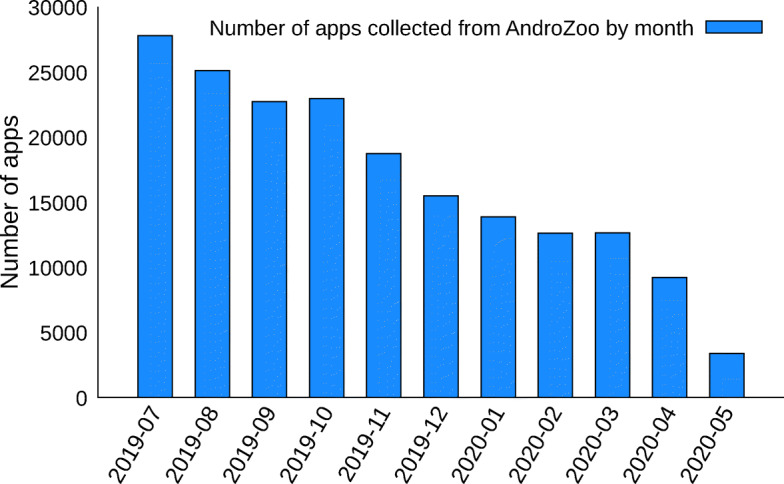
Monthly distribution of Android apps considered in this study

Since the COVID-19 outbreak is quite recent, we did not consider the 11 million Android apps present in AndroZoo. For our study, we instead considered and collected from AndroZoo all the apps ranging from July, 1^st^ 2019 to May, 25^th^ 2020, leading to a total number of 184432 collected apps. Note that AndroZoo does not contain information about the release date of an app, i.e., there is no information about when an app has been uploaded on the market. For this reason, we approximate the date of an app by considering the date of the dex files in apks. Figure 1 shows the distribution of collected APKs according to the month of the date of the dex files in APKs.

### Dataset Augmentation

It is a known problem that the date metadata in APKs (approximated as the date of the dex files) is not always reliable (Li et al. 2018). For this reason, we decided to augment our dataset (1) by not considering the time window from July 2019 to May 2020 and (2) by selecting any apps from AndroZoo whose package name contained the words *covid-19*, *covid19* or *coronavirus*. With this heuristic, we were able to retrieve 131 apps from AndroZoo.

⇒ Finally, our initial dataset is constituted of 184563 Android apps.

### Short Background on Android Apps

In general, Java is the language used by developers to create Android applications, though other languages can be used such as Kotlin, or even languages such as C and C++ thanks to the Native Development Kit (NDK). For common Java applications, the source code is compiled into Java bytecode and run into the Java virtual machine. However, Android apps are compiled into Dalvik bytecode (Dalvik Executable, i.e., Dex), which is the distribution format of app code in app packages. This bytecode is then targeted to be executed on the Android Android Runtime (which now replaces Dalvik Virtual Machine on recent Android versions).

Still, the DEX files do not constitute an application. Indeed, an Android app is a collection of files packaged together in the so-called Android Application Package (APK). This format is used for distributing apps on devices, not only via official and unofficial markets but also via any other channels where an independent file can be distributed. Indeed, Android devices allow users to install applications from any sources once a configuration option is toggled off.

An APK is typically a zip file containing the following files: (1) Metadata files, (2) the certificate(s) used to sign the application, (3) a lib folder containing platform-dependent compiled code, (4) compiled and non-compiled resource files, (5) one or multiple DEX files, generally named classes*X*.dex with *X* an integer, (6) an AndroidManifest.xml file describing the application (package name, components, version, access rights, etc.).

## Experiment Setup

In this section, we describe the setup of our experiments. More specifically, in Section 3.1 we describe how we create a dataset of Covid-related apps by searching apps in both our initial dataset extracted from AndroZoo, and on the web. Then we describe in Section 3.3 how the information needed for answering the research questions was extracted.

### Dataset Curation

The large majority of apps contained in our initial dataset of Android apps are not related to the COVID-19 outbreak. Consequently, we need to curate this dataset. Our first idea was to rely on a clear definition of what is a Covid-related app. However, we realized that finding a correct and precise definition is not obvious. For instance, the english Wikipedia page^1^ related to COVID-19 (Wikipedia 2020) defines Covid-related apps as *“mobile software applications that use digital tracking to aid contact tracing in response to the COVID-19 pandemic, i.e. the process of identifying persons (“contacts”) who may have been in contact with an infected individual.”*. We quickly considered this definition as too restrictive since we found several Covid-related apps that are not about “digital contact tracing”. As a result, rather than relying on a definition of what is a Covid-related app we (1) implemented several heuristics based on assumptions, and (2) performed several quality checks to filter out irrelevant apps.

#### **Assumption 1**

Our first assumption is that a Covid-related app contains *strings* (e.g., in a class name or method name) related to COVID-19. Based on this assumption, we defined several regular expressions that we apply on various fields on an Android app. More specifically, we proceed as follows: Let *A* be a set of Android apps and *apk* an app of this set. Let be:

- *C*_*apk*_ the set of classes names in *apk*,
- *M*_*apk*_ the set of method names in *apk*,
- *F*_*apk*_ the set of file names in *apk*,
- *S*_*apk*_ the set of strings contained *apk*,

We keep *apk* if at least one element of *C*_*apk*_ ∪ *M*_*apk*_ ∪ *F*_*apk*_ ∪ *S*_*apk*_ matches at least one regular expressions listed in Table 1. An app is kept if for instance, a class name contains the substring *coronavirus*, or if the app contains a string with *pandemi* as substring.

**Table 1:** Regular expressions used to filter out non Covid-related apps

“(?i).*coronavirus”	“(?i).*corona”
“(?i).*sars(-?cov)”	“(?i).*quarantin.*”
“(?i).*lock-?down”	“(?i).*containment”
“(?i).*social-?distanc.*”	“(?i).*pandemi.*”
“(?i).*out-?break”	“(?i).*epidemi.*”
“(?i).*confinement”	

**Fig. 2:**
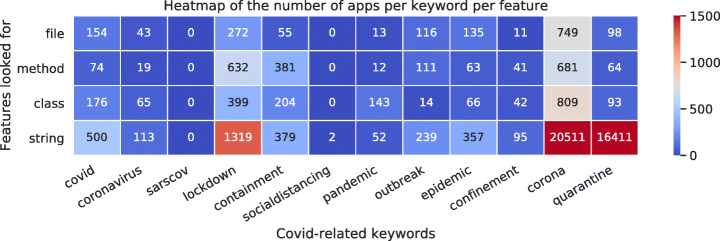
Heatmap representing the number of apps gathered by each Covid-related keyword, and for each family of features

We ended up with a set of 35613 supposedly Covid-related apps. In Fig. 2, we can see the number of apps that were retrieved per keyword (note that several keywords can be present in a given app).

**Quality Check #1:** We can see in Fig. 2 that the keywords *corona* and *quarantine* match a significantly larger number of apps. After investigation, we found that the keyword *quarantine* is used in strings by developers to check if the smartphones are *rooted*, i.e., if the user has super-user (*root*) privilege. Indeed, the presence of some packages can characterize the fact that a smartphone is rooted. Among such packages, one is named com.ramdroid.appquarantine and another is named com.ramdroid.appquarantinepro. To verify the presence of these two packages, developers often use these package names as string, and thus the regular expression using *quarantine* matches. This explains why there is such a high number of apps containing *quarantine* in strings. We manually analyzed several dozens of apps and confirmed they are not real Covid-related apps (mainly music, education, news, shopping, and game apps).

Regarding the *corona* keyword, it also appears in a substantial number of apps, especially in string feature. After manual investigation, we found that apps retrieved with this keyword use a framework called *Corona* developed by Coronalabs.^2^ These apps are mainly games, entertainment and personalization apps. Moreover, the word *corona* not only refers to COVID-19, but also has uses in several unrelated contexts (in architecture, beverages, books, music, movies, and games).

We decided to rule out both keywords *corona* and *quarantine*, as they revealed to mostly bring noise in our dataset. After filtering the apps gathered, i.e., not taking into account the *corona* and *quarantine* keywords, we obtained 4103 apps.

#### **Assumption 2**

Our second assumption is that since AndroZoo is known to contain successive versions of the same apps (Allix et al. 2016; Li et al. 2018), our dataset contains successive versions of the same Covid-related app. Therefore, for the subsequent analyses, we only keep the latest version of a given app. This step is performed by comparing apps version code—available in AndroZoo metadata—and keeping the highest value. After this step, our dataset contains 750 apps.

#### **Assumption 3**

**Fig. 3:**
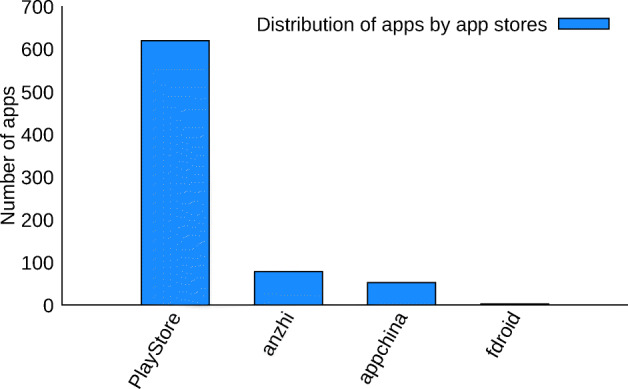
Distribution of the 750 supposedly Covid-related apps by markets where they were obtained from

Our third assumption is that *official* Covid-related apps are released only on the official Android market, i.e., the Google Play market. Figure 3 depicts the distributions of the apps per market from where they have been crawled. Most of the apps were released in the official Google Play market. By considering apps from Google Play only, we reach the number of 619 apps.

**Fig. 4:**
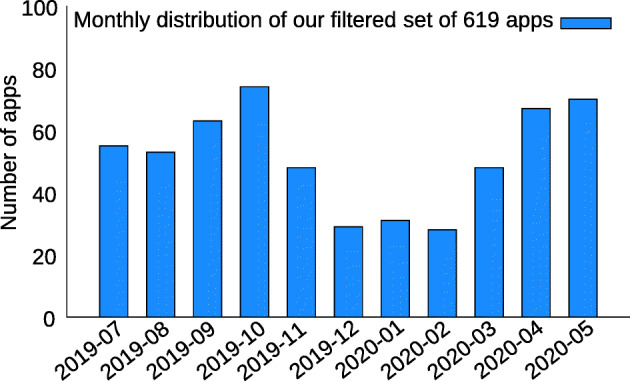
Timeline representing the number of apps with Covid-related keywords in function of time

**Quality Check #2:** To check the quality of our dataset, we inspect the date of the dex file in the APKs. The COVID-19 outbreak was not known until the very end of 2019, so we expect to find apps from 2020 only. Figure 4 presents the distribution of those apps by the date of the dex file in the APK. First, we observe that fewer apps were released during winter 2019-2020. Then we can see that starting from March 2020, a significant amount of apps were developed, which corresponds to the containment period.

Finally, Fig. 4 shows that our set of Covid-related apps contains a significant amount of apps developed before the pandemic, which indicates the low quality of our dataset and that an additional layer of filtering is needed to only keep Covid-related apps. Indeed, our manual investigations of those apps revealed that they contained keywords that may be overly broad. For example, it appeared that keywords such as *outbreak*, *pandemic*, *containment* or *lockdown* were popular in games, most notably in the zombies and survival games genres.

#### **Assumption 4**

Our fourth assumption is that the description of an app is a reliable source of information to check that an app is Covid-related. AndroZoo does not provide the app descriptions, so we queried Google Play to retrieve the description of the 619 apps. Note that it was possible to get the description for 537 apps, while 82 apps were not available anymore in Google Play at that time. Actually, AndroZoo uses a crawler to automatically download apps. It is possible that AndroZoo downloads an app at a time *t*_1_, but if Google Play decides to remove this app (or if the developer decides to remove it) at time *t*_2_, with *t*_1_ < *t*_2_, it is not possible to access the description of the app anymore after time *t*_2_. We found that Google is actively deleting apps that are violating their policy with respect to COVID-19 (Carman 2020; Google 2020a).

Manual investigations of the remaining 537 apps were conducted to qualify an app as *Covid-related*. We did so by analyzing the Google Play page of every app, reading the description, and looking at screenshots. We did not encounter any ambiguous case, hence it was straightforward to qualify an app as Covid-related or not. With this method, we determined with high certainty that 44 apps taken from AndroZoo were Covid-related.

Note that the set of apps from our initial dataset for which the package name contained Covid-related keywords contained 131 elements and we only retrieved 44 apps. This is explained by the fact that among those 131 apps, there were, for almost one-fifth, different versions of the same app. In addition, we were not able to analyze the Google Play page of apps that were not available in the Google Play market.

**Fig. 5:**
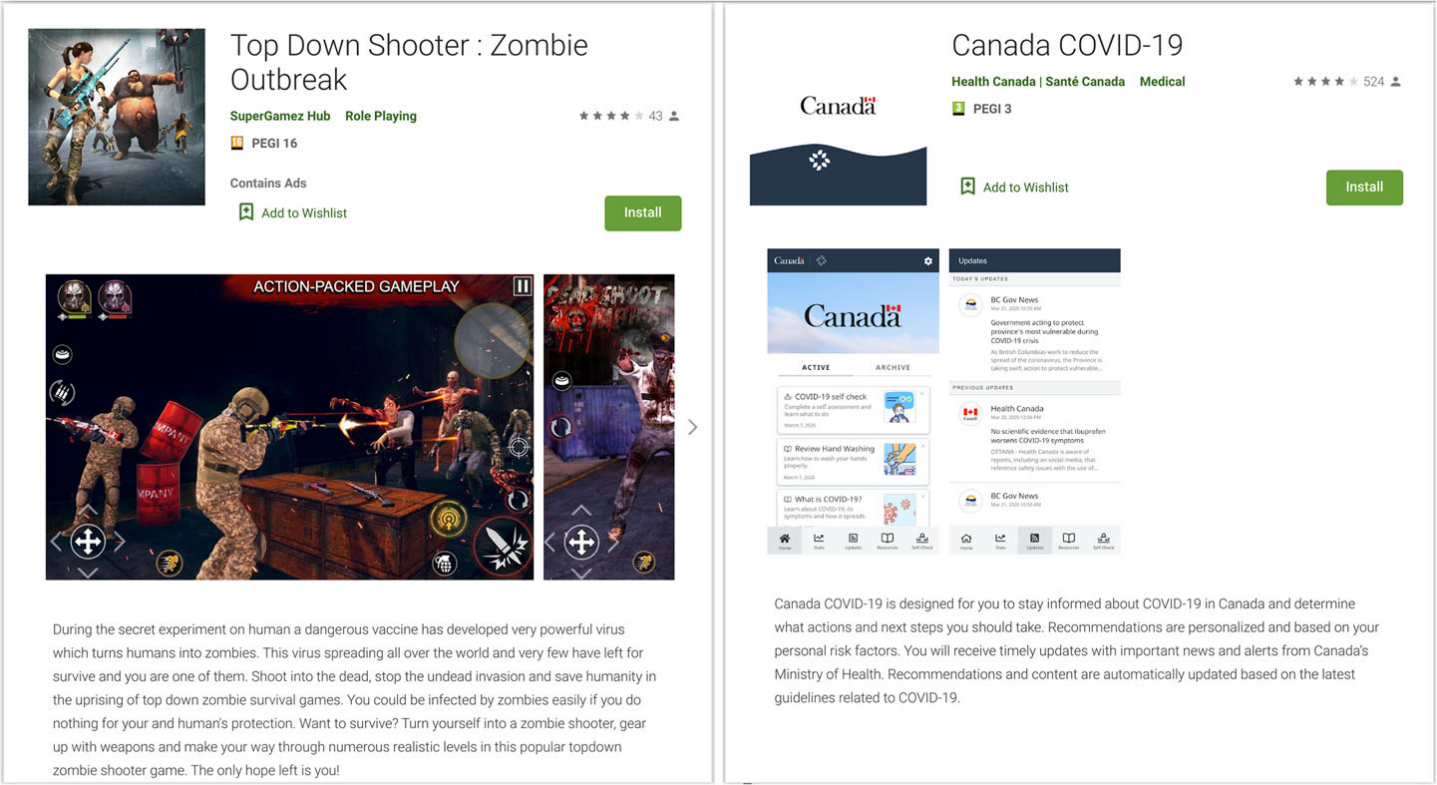
Comparison between two Google Play page of two different apps. Left app: Zombie game; right app: Covid-related app

Figure 5 gives two examples to show an app we discarded and an app that was considered as Covid-related. First, the game picture on the left clearly shows a game in which the user has to kill zombies, even though the game contains the string *outbreak*. Second, the Covid-related app on the right is explicit regarding the content delivered to the user. The title refers to COVID-19, the description gives clues about the content, i.e., information about COVID-19 and guidelines about COVID-19. Besides, the screenshots are also explicit by depicting what actions users can perform, here Covid-related actions, i.e., getting information about COVID-19, performing self-diagnosis, receiving guidelines, and news about COVID-19. It represents how unequivocally our decisions were to be made to qualify an app as Covid-related or not.

#### **Assumption 5**

As AndroZoo is not exhaustive and all the apps in the Google Play market are not available for download from every country (AndroZoo crawls from specific countries), we felt the need to expand our research. Consequently, manual investigations were conducted on the web to search for apps that would not be available in Google Play and/or that our empirical analysis on AndroZoo did not catch. We found 48 additional apps from diverse countries from different time span, e.g. we found apps that were first seen in June 2020.

We verified if any of those 48 additional Covid-related apps were already in Androzoo and might have been missed by our diverse filters, it was not the case for all of them. Thus, our set of COVID-applications reached a length of Covid-related apps, each one of them from Google Play.

**Fig. 6:**
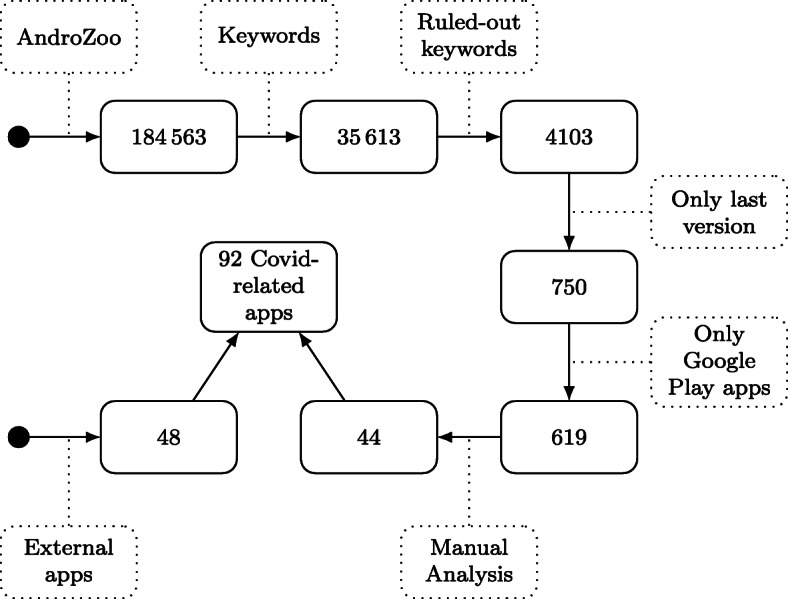
Process of our dataset curation. Numbers represent numbers of apps. Dotted boxes represent filters used to refine the dataset

⇒ Figure 6 summarizes our dataset curation process.

### When Did Covid-Related Apps Start to Appear in Google Play?

**Fig. 7:**
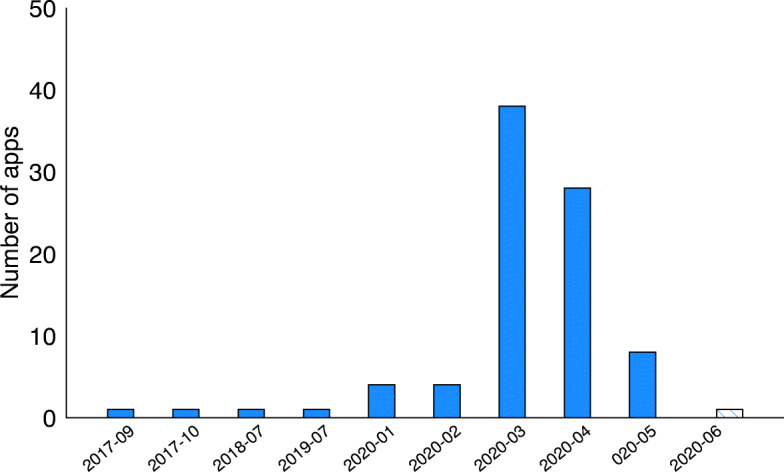
Number of Covid-related apps by appearance date. The month of June appears hatched to represent the fact that our study was conducted in early June, which means we do not have all apps released in June

In this section, we consider the 92 collected Covid-related apks. As the first release date of apps is not available on Google Play, we need to rely on other sources of information to try to find the first appearance date of a given app. Therefore, in order to visualize when Covid-related apps appeared, we considered, for each app (when available), the earliest date among the AndroZoo added date, the first seen dates in VirusTotal and in third party datasets (i.e., Koodous,^3^ APKCombo,^4^ APKPure,^5^ and AppBrain).^6^ The results can be seen in Fig. 7.

First we can see that four apps were released long before 2020 (com.intelligent.alertaguate, br.gov.datasus.guardioes, pl.nask.mobywatel and co.gov.ins.guardianes), i.e., two in 2017, one in 2018 and one in 2019. After investigation, we found that these apps were originally developed as governmental health apps and were updated to account for the pandemic specificities.

In 2020, we can see that a few Covid-related apps started to appear as early as January 2020, i.e., before the pandemic was officially recognized by the World Health Organization (i.e., March, 11^th^ 2020) (Ghebreyesus 2020).

We also note from Fig. 7 that in March 2020, the number of new apps increased drastically. Finally, since we stopped the collection process in early June 2020 (prior to submission), only a few Covid-related apps have been collected this month. Our artefact of collected apps however has been updated with new apps that appeared after our submission. Evidently, these apps could not be taken into account in our empirical analysis.

### Features Extraction

In this section, we expose what features were extracted from the apps considered in this study and how we extracted it. Three ways of retrieving the needed information were considered: (1) Extracted from AndroZoo, (2) Automated analysis of the apps, and (3) Manual analysis, which we describe below.

#### AndroZoo Metadata

Apps from AndroZoo are provided with additional metadata, i.e., a vector of length 11 representing different information. Those metadata elements are used to expose several properties of the apps under analysis. We consider the date of the dex files to verify when the Covid-related apps started to appear. The package names and version codes were useful to have some insights into the versioning of the apps and to keep only the latest version of the app considered, i.e., one version of the app will be analyzed. When available, the number of AntiVirus products reporting an app as malicious (obtained from VirusTotal) was used for qualifying the maliciousness of the apps. Finally, AndroZoo metadata also indicate the source where an app was obtained from.

#### Automated Analyses of Apps

AndroZoo metadata being limited, we additionally leveraged existing tools and frameworks to analyze Android apps in order to obtain the information to acquire the information needed by our study.

- We developed a program relying on the Androguard software package.^7^ Thanks to this tool, we extract the permissions requested by the apps, as well as information about components in the applications (i.e., Activities, Services, etc.). We also automatically compute the complexity metrics described in Appendix A.
- We used CogniCrypt (Krüger et al. 2017) and its headless implementation CryptoAnalysis (CryptoAnalysis 2020) to check that they were written following best practices regarding cryptographic APIs.
- As several Covid-related apps seek to acquire and process the location of users (considered a piece of sensitive information), we verify if those apps were subject to data leaks. Hence, we leverage FlowDroid-IccTA (Li et al. 2015), the state-of-the-art static data leak detector dedicated to Android apps.

#### Manual Analyses of Apps

While application information that can be readily obtained through automatic tools were necessary for our study, we went a step further and acquired *qualitative* data on the apps.

By collecting and carefully reading the descriptions of apps^8^ and by confirming the validity of our understanding by matching the reviews and app screenshots with the description, we were able to assemble highly-qualified data on the goals of app developers, on whether an app is developed by a state body or an individual person, etc.

Finally, after leveraging the automated tools described above, their results were manually confirmed for the set of Covid-related apps (i.e., 92). This allowed us to ensure those tools did not yield false-positives, and that their results were sound and consistent.

In the following, we give further details on how the manual analyses were performed:

- App descriptions: The first author read each app description systematically extracting relevant information on the WHO, WHAT, WHEN and HOW that characterize the apps. To ensure that the collected information is reliable, the extraction is repeated for every app. Then he proposed a first summary based on a careful analysis of recurrence in characteristics. The author team subsequently convened to refine this categorisation and validate the final taxonomy.
- App properties: In order to verify some properties on apps, the first author decompiled each app (using JADX)^9^ and searched for the property to verify its use (e.g., is a certain keyword used in the context of a game or is the app really providing information or service about to Covid?)
- Tool results: The first author ran the third-party tools multiple times on Covid-related apps. For instance, every output of IccTA was manually checked to confirm that it is not a false positive.

## Results

In this section, we present our experimental results and we answer our research questions.

### What are Covid-Related Apps Used for?

#### Motivation

The sudden increase of Covid-related apps during the pandemic shows that Android apps developers have been active in providing end-users with solutions to address the COVID-19 pandemic. Nevertheless, the functionalities of Covid-related apps are not known and have not been studied. In this section, we study, characterize, and build categories from which Covid-related apps belong. The categorization of Covid-related apps offers a first layer of knowledge toward understanding them. The outcome of this research question will give an overview of Covid-related apps’ functionalities to the general public.

#### Strategy

Textual descriptions of apps on markets generally provide a wealth of information on the purpose and functionalities that developers advertise. We undertake to systematically examine the descriptions of all the apps under study. Unfortunately, since Google Play is actively moderating Covid-related apps, we have faced an issue with some apps that we were able to initially collect but which were no longer available on the market at the time of analysis. Eventually, our analysis of descriptions was performed on 78 apps. In other words, from the time we read the descriptions of the apps to curate our dataset (as explained in Section 3.1) and the time we perform this more in-depth study, i.e., collecting information related to the features of the apps, 14 apps (92 − 78) were not able anymore on GooglePlay.

#### A Taxonomy of Covid-Related Apps

After a careful analysis of information available in Google Play, we summarize for each app its general goal, i.e., which aspects of the COVID-19 crisis the app is precisely intended to address. Eventually, we identified three main categories to which each app can be associated with possible overlap between categories, i.e., an app can be associated with several categories:

1. *Information broadcast (top-down)* - Apps in this category aim to provide users with various types of information, from general guidelines, infection statistics to general COVID-19 news. Although such apps are not always officially released by government bodies, they often relay official information from top (authorities) down (users).
2. *Upstream collection (bottom-up)* - Apps in this category collect information from users and make it available to the developer and/or an official body, such as a country’s health authorities.
3. *Tooling* - Apps in this category serve as tools with functionalities that directly deal with daily aspects of the COVID-19 (e.g., generation of certificates).

#### [1] Information broadcast

**Fig. 8:**
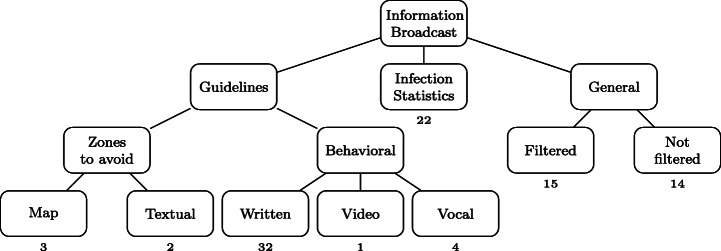
Information Broadcast category. The number below a leaf box indicates the number of related apps

From the collected dataset of Covid-related apps we identified several distinguishing scenarios in apps performing information broadcasting. Figure 8 overviews the related characteristics, notably based on the types of information that are made available to the user:

1. *Guidelines* on measures to take to minimize the risk of infection - Among such apps, some render maps highlighting high-risk areas. Other apps provide behavioral advice (e.g., how to wash hands), leveraging the whole spectrum of available media: (1) textual descriptions (for the majority of apps), (2) videos and, (3) audio clips.
2. Continuously-updated *Statistics* on the pandemic evolution;
3. *General information* about COVID-19, such as about the typical symptoms. We identified two different scenarios in the provision of general information:
  Some apps present *curated information*, i.e., information that is somehow checked and filtered by the development team before it is shown to the public. Such information is often tagged in a way that allows interested people to find the source, and gauge its credibility. Sometimes, these apps are developed directly by an entity that itself carries credibility as a source of information, such as national healthcare authorities. An example is the *MyHealth Sri Lanka* app^10^ developed by the national ICT Agency, which presents to the user *verified information on the current COVID-19 status.*
  A number of apps appear to provide unfiltered information regarding COVID-19. Their developers are not always themselves entities that would traditionally be assumed to have any specific credibility on the matter. For example, the DiagnoseMe app,^11^ which claims to provide the user with *all the information on the virus*, is proposed by an association with unrecorded expertise in health.

#### [2] Upstream Collection

Most apps in our dataset perform data collection from users. This suggests that many app providers consider *data* to be key in the mitigation of the COVID-19 crisis. App providers indeed collect a variety of information, including user personal information (e.g., name, age, address, etc.), some medical information (e.g., whether a user is infected with COVID-19, the therapies that are used). Some apps are even used to keep a health diary (sharing information about symptoms every day), or to report the infection of people in the app user’s acquaintances.

**Fig. 9:**
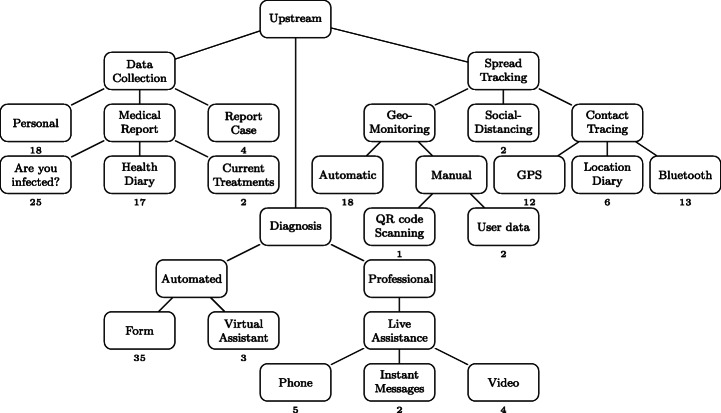
Upstream category

Overall, we have identified three different ways in which apps collect user data, as summarized in Fig. 9. Note that in the case of *data collection* and *spread tracking* apps, we did not try to qualify whether apps were as privacy-preserving as their developers claimed they were (e.g., data is deleted after *N* days), nor to determine to what extent the collected data is shared with third parties.

Similarly, for this paper, we did not analyze the inner workings of contact-tracing apps, and we did not evaluate the merit nor the opportunity of contact-tracing, this having already been—and still being to this date—discussed by security researchers (Culnane 2020; Anderson 2020; Baumgärtner et al. 2020).

Several apps take inputs from the users to offer diagnoses related to COVID-19. Such apps can provide a built-in questionnaire that users have to fill within the app, or leverage a virtual assistant or chatbot. In these cases, the diagnosis can be made automatically, with no interaction nor confirmation with a trained medical practitioner.

Other apps, however, provide a somewhat more traditional medical visit experience, by offering the facilities needed to remotely exchange (e.g., via instant text messages as well as voice and/or video calls) with a medical doctor. Such apps are used from home, since millions of people worldwide were confined, and were potentially reluctant or unable to visit a brick-and-mortar doctor’s office.

Additionally, some apps are developed to track the spread of the virus by locating the users of the apps. While a few of those apps use simple geo-monitoring with GPS information for tracking users, most apps do it automatically. Nevertheless, we found a few apps that request users to provide a-posteriori the locations they have visited on a given day. We also identified one app which uses QR code scanning at the entrance of public buildings to obtain precise location information, while still being fully under users’ control.

With respect to tracing, a few apps promote social-distancing using the GPS location of users, the goal being to not approach other people too closely.

Furthermore, several apps implement contract-tracing, i.e., the ability to retrieve who a specific person has been in contact with, providing users a way to know if they have encountered someone infected, and potentially infectious. Contact-tracing apps mainly rely on three methods, (1) Using the GPS location of users, (2) Using the Bluetooth technology to detect proximity, and (3) Using a location diary that the users have to manually fill.

#### [3] Tooling

**Fig. 10:**
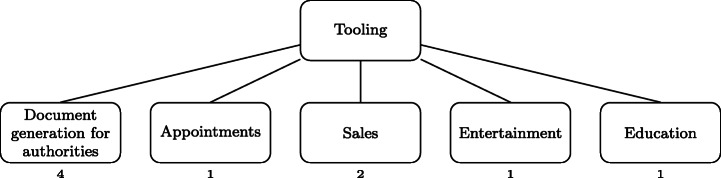
Tooling category

The last category is the *tooling* category which includes several types of tools aimed at helping users deal with some consequences of the COVID-19 crisis (see Fig. 10). A few apps allow users to auto-generate documents for their local authorities (e.g., travel authorization that had been made mandatory in several countries during containment).

Users can also install apps offering appointment-capabilities for medical purposes, or selling Covid-related products (e.g., masks, hand-sanitizers, etc.).

On the entertainment front, apps were released proposing games around the pandemic, or providing users with COVID-19-themed image filters, for example adding a virtual mask, or adding virtual decorative elements to an actual mask.

Lastly, apps were also made to cater to the newly-discovered needs of massive remote education.

The interested reader can inspect Tables 2, 3 and 4 for more information about the mapping between categories and the apps for which we were able to retrieve the relevant information.

**Table 2:** First part of Covid-related apps’ characteristics retrieved from Google Play apps pages

Package name	Country	Developer type	Target	Information Broadcast							
				General Information Filtered	Info not filtered	Vocal Guidelines	Video Guidelines	Written Guidelines	Map Zones To Avoid	Sales	Textual Zones to Avoid
com.gov.mcmc.projectcatur	Malaysia	Governmental	Citizen	✓				✓			
com.mohw.corona	South Korea	Governmental	Foreigners								
kg.cdt.stopcovid19	Kyrgyztan	Governmental	Citizen								
pl.nask.mobywatel	Poland	Governmental	Citizen	✓				✓			
app.ceylon.selftrackingapp	Sri Lanka	Governmental	Citizen	✓							
bf.diagnoseme.fasocivic	Burkina Faso	Association	Citizen		✓	✓	✓	✓	✓	✓	
com.moc.gh	Ghana	Governmental	Citizen						✓		
com.vost.covid19mobile	Portugal	Governmental	Citizen		✓			✓			
sg.gov.tech.bluetrace	Singapore	Governmental	Citizen					✓			
com.joinzoe.covid_zoe	UK/Sweden	Company	Citizen								
am.gov.covid19	Armenia	Governmental	Citizen								
sa.gov.nic.tawakkalna	Saudi Arabia	Governmental	Citizen								✓
fr.gouv.android.stopcovid	France	Governmental	Citizen					✓			
is.landlaeknir.rakning	Iceland	Governmental	Citizen								
jo.gov.moh.aman	Jordan	Governmental	Citizen					✓			
nz.govt.health.covidtracer	New Zealand	Governmental	Citizen								
es.gob.asistenciacovid19	Spain	Governmental	Citizen		✓						
com.moi.covid19	Qatar	Governmental	Citizen		✓						
au.gov.health.covid19	Australia	Governmental	Citizen	✓				✓			
at.roteskreuz.stopcorona	Autriche	NGO	Citizen								
com.proudcrowd.care	USA	Company	Citizen								
com.ri.crushcovid	USA	Governmental	Citizen		✓						
au.gov.health.covidsafe	Australia	Governmental	Citizen					✓			
ca.bc.gov.health.hlbc.COVID19	Canada	Governmental	Citizen	✓				✓			
de.kreativzirkel.coronika	Germany	Company	Citizen					✓			
com.coronacheck.haugxhaug.testyourcorona	Germany	Researchers	Citizen	✓				✓			
cat.gencat.mobi.StopCovid19Cat	Spain	Governmental	Citizen					✓			
cat.gencat.mobi.confinApp	Spain	Governmental	Citizen	✓							
com.ambulis.aphm.covid	France	Company	Patients					✓			
fr.aphp.covidom	France	Hospital	Patients								
appinventor.ai_david_taylor .Coronavirus_help2020	Worldwide	Independent	Citizen		✓						
uy.gub.salud.plancovid19uy	Uruguay	Governmental	Citizen								
com.covid19_algeria	Algeria	Governmental	Citizen		✓						
com.agetic.coronavirusapp	Bolivia	Governmental	Citizen	✓				✓			
ch.covid19bs.app.PMSMobile	Switzerland	Governmental	Citizen					✓			
es.gva.coronavirus	Spain	Governmental	Citizen		✓						
com.govpk.covid19	Pakistan	Governmental	Citizen								
covid19care.virus.coronavirus .corona.sick.marcom.health.pakistan	Pakistan	Company	Media Journalists		✓			✓			
com.tommasomauriello .autocertificazionecoronavirus	Italia	Independent	Citizen								
ca.gc.hcsc.canada.covid19	Canada	Governmental	Citizen	✓	✓			✓			
com.krrsoftwaresolutions11.Facemasks	Undefined	Company	Consumers							✓	
it.adilife.covid19.app	Italia	Company	Citizen								
it.adl.aslroma3.covid19.app	Italia	Company	Citizen								
com.coronavirus.facemask	Undefined	Company	Users								
com.osapps.covid19	Undefined	Company	Citizen		✓			✓			
com.maithu.transplantbuddy.covid19	Ireland	Company	Patients								
ar.gob.coronavirus	Argentina	Governmental	Citizen								
com.bloomreality.sodi	Undefined	Company	Citizen								
br.gov.datasus.guardioes	Brazil	Governmental	Citizen	✓				✓	✓		
cz.covid19cz.erouska	Czech Republic	Governmental	Citizen					✓			
com.covid19.dgmup	India	Governmental	Citizen					✓			
com.docandu.checker	Greece	Company	Citizen					✓			
it.softmining.projects.covid19 .savelifestyle	Italia	Company	Citizen			✓					
nic.goi.aarogyasetu	India	Governmental	Citizen								✓
com.dawsoftware.contacttracker	Undefined	Company	Citizen								
com.hamagen	Israel	Governmental	Citizen								
hu.gov.virusradar	Hungary	Governmental	Citizen								
world.coalition.app	Undefined	Company	Citizen								
mk.gov.koronavirus.stop	Macedonian	Governmental	Citizen								
org.sshield.selfshield	Common Weath	Association	Citizen								
org.pathcheck.covidsafepaths	Undefined	Association	Citizen								
com.knasirayaz.mohapcovid	United Arab Emirates	Governmental	Citizen	✓				✓			
gov.georgia.novid20	Georgia	Governmental	Citizen								
nl.lumc.covidradar	Netherlands	Governmental	Citizen		✓			✓			
com.Eha.covid_19	Vietnam	Governmental	Citizen		✓	✓		✓			
covid.trace.morocco	Morocco	Governmental	Citizen					✓			
ru.mos.socmon	Russia	Governmental	Patients					✓			
org.prixa.p5covidtracker	Nepal	Governmental	Citizen	✓							
de.bssd.covid19	Germany	Company	Patients								
com.edu.aku.akuhccheck	Pakistan	Researchers	Citizen	✓		✓		✓			
com.pixxonai.covid19	India	Governmental	Quarantine persons								
de.rki.coronadatenspende	Germany	Governmental	Citizen								
mx.gob.www	Mexico	Governmental	Citizen	✓				✓			
tr.gov.saglik.koronaonlem	Turkey	Governmental	Citizen					✓			
com.telkom.tracencare	Indonesia	Governmental	Citizen								
co.gov.ins.guardianes	Colombia	Governmental	Citizen	✓							
bh.bahrain.corona.tracker	Bahrain	Governmental	Citizen					✓			
bg.government.virusafe	Bulgaria	Governmental	Citizen		✓

**Table 3:** Second part of Covid-related apps’ characteristics retrieved from Google Play apps pages

Package name	Upstream										
	Health Diary	QR Code Tracing	Automated Geo-Monitoring	Are You Infected?	Report Covid Case	GPS Contact Tracing	Automatic Diagnosis	Live-Assistance Chat	Bluetooth Contact Tracing	Personnal Data Collection	Current Treatment
com.gov.mcmc.projectcatur						✓					
com.mohw.corona	✓										
kg.cdt.stopcovid19			✓	✓	✓						
pl.nask.mobywatel											
app.ceylon.selftrackingapp			✓								
bf.diagnoseme.fasocivic							✓	✓			
com.moc.gh							✓				
com.vost.covid19mobile											
sg.gov.tech.bluetrace									✓		
com.joinzoe.covid_zoe	✓			✓			✓			✓	✓
am.gov.covid19							✓				
sa.gov.nic.tawakkalna					✓		✓				
fr.gouv.android.stopcovid				✓					✓		
is.landlaeknir.rakning			✓	✓		✓					
jo.gov.moh.aman			✓	✓							
nz.govt.health.covidtracer		✓								✓	
es.gob.asistenciacovid19			✓				✓			✓	
com.moi.covid19			✓							✓	
au.gov.health.covid19							✓				
at.roteskreuz.stopcorona				✓			✓		✓		
com.proudcrowd.care				✓		✓					
com.ri.crushcovid			✓	✓		✓	✓				
au.gov.health.covidsafe				✓	✓				✓		
ca.bc.gov.health.hlbc.COVID19						✓				✓	
de.kreativzirkel.coronika	✓										
com.coronacheck.haugxhaug .testyourcorona							✓				
cat.gencat.mobi.StopCovid19Cat	✓			✓			✓				
cat.gencat.mobi.confinApp											
com.ambulis.aphm.covid	✓						✓				
fr.aphp.covidom	✓						✓				
appinventor.ai_david_taylor.Coronavirus_help2020											
uy.gub.salud.plancovid19uy	✓						✓			✓	
com.covid19_algeria			✓							✓	
com.agetic.coronavirusapp							✓				
ch.covid19bs.app.PMSMobile				✓			✓				
es.gva.coronavirus							✓				
com.govpk.covid19											
covid19care.virus.coronavirus.corona.sick.marcom.health.pakistan					✓						
com.tommasomauriello.autocertificazionecoronavirus										✓	
ca.gc.hcsc.canada.covid19							✓				
com.krrsoftwaresolutions11.Facemasks											
it.adilife.covid19.app	✓						✓			✓	
it.adl.aslroma3.covid19.app	✓						✓			✓	
com.coronavirus.facemask											
com.osapps.covid19											
com.maithu.transplantbuddy.covid19	✓		✓				✓				✓
ar.gob.coronavirus			✓				✓				
com.bloomreality.sodi											
br.gov.datasus.guardioes							✓				
cz.covid19cz.erouska				✓					✓		
com.covid19.dgmup										✓	
com.docandu.checker							✓	✓			
it.softmining.projects.covid19 .savelifestyle						✓	✓				
nic.goi.aarogyasetu				✓					✓		
com.dawsoftware.contacttracker	✓					✓	✓				
com.hamagen			✓	✓			✓				
hu.gov.virusradar						✓			✓		
world.coalition.app				✓					✓		
mk.gov.koronavirus.stop				✓					✓		
org.sshield.selfshield	✓						✓				
org.pathcheck.covidsafepaths				✓		✓					
com.knasirayaz.mohapcovid											
gov.georgia.novid20				✓		✓			✓		
nl.lumc.covidradar										✓	
com.Eha.covid_19			✓								
covid.trace.morocco				✓					✓		
ru.mos.socmon			✓							✓	
org.prixa.p5covidtracker	✓			✓			✓			✓	
de.bssd.covid19				✓							
com.edu.aku.akuhccheck							✓				
com.pixxonai.covid19	✓		✓				✓			✓	
de.rki.coronadatenspende	✓										
mx.gob.www							✓				
tr.gov.saglik.koronaonlem			✓				✓			✓	
com.telkom.tracencare				✓					✓		
co.gov.ins.guardianes	✓		✓	✓			✓			✓	
bh.bahrain.corona.tracker			✓	✓		✓			✓	✓	
bg.government.virusafe	✓		✓	✓		✓	✓

**Table 4:** Third part of Covid-related apps’ characteristics retrieved from Google Play apps pages

Package name	Upstream						Tooling				
	Live-Assistance Phone	User data Geo-Monitoring	Location Diary	Virtual Assistant	Live-Assistance Video	Social-Distancing	Appointment	Entertainment	Sales	Education	Document Creation
com.gov.mcmc.projectcatur											
com.mohw.corona											
kg.cdt.stopcovid19											
pl.nask.mobywatel											
app.ceylon.selftrackingapp											
bf.diagnoseme.fasocivic									✓	✓	
com.moc.gh											
com.vost.covid19mobile											
sg.gov.tech.bluetrace											
com.joinzoe.covid_zoe											
am.gov.covid19											
sa.gov.nic.tawakkalna											✓
fr.gouv.android.stopcovid											
is.landlaeknir.rakning											
jo.gov.moh.aman											
nz.govt.health.covidtracer			✓								
es.gob.asistenciacovid19											
com.moi.covid19	✓										
au.gov.health.covid19											
at.roteskreuz.stopcorona											
com.proudcrowd.care		✓	✓								
com.ri.crushcovid			✓								
au.gov.health.covidsafe											
ca.bc.gov.health.hlbc.COVID19											
de.kreativzirkel.coronika		✓									
com.coronacheck.haugxhaug .testyourcorona											
cat.gencat.mobi.StopCovid19Cat											
cat.gencat.mobi.confinApp				✓							✓
com.ambulis.aphm.covid											
fr.aphp.covidom	✓										
appinventor.ai_david_taylor .Coronavirus_help2020											
uy.gub.salud.plancovid19uy					✓						
com.covid19_algeria											
com.agetic.coronavirusapp											
ch.covid19bs.app.PMSMobile											
es.gva.coronavirus							✓				
com.govpk.covid19											
covid19care.virus.coronavirus .corona.sick.marcom.health.pakistan											
com.tommasomauriello .autocertificazionecoronavirus											✓
ca.gc.hcsc.canada.covid19											
com.krrsoftwaresolutions11.Facemasks									✓		
it.adilife.covid19.app					✓						
it.adl.aslroma3.covid19.app					✓						
com.coronavirus.facemask								✓			
com.osapps.covid19											
com.maithu.transplantbuddy.covid19											
ar.gob.coronavirus											✓
com.bloomreality.sodi						✓					
br.gov.datasus.guardioes											
cz.covid19cz.erouska	✓										
com.covid19.dgmup			✓								
com.docandu.checker											
it.softmining.projects.covid19.savelifestyle											
nic.goi.aarogyasetu											
com.dawsoftware.contacttracker											
com.hamagen											
hu.gov.virusradar											
world.coalition.app											
mk.gov.koronavirus.stop											
org.sshield.selfshield											
org.pathcheck.covidsafepaths			✓								
com.knasirayaz.mohapcovid	✓										
gov.georgia.novid20											
nl.lumc.covidradar											
com.Eha.covid_19				✓	✓						
covid.trace.morocco											
ru.mos.socmon											
org.prixa.p5covidtracker											
de.bssd.covid19											
com.edu.aku.akuhccheck				✓		✓					
com.pixxonai.covid19											
de.rki.coronadatenspende											
mx.gob.www											
tr.gov.saglik.koronaonlem			✓								
com.telkom.tracencare	✓										
co.gov.ins.guardianes											
bh.bahrain.corona.tracker											
bg.government.virusafe											

Table 2 gives the list of the 78 Covid-related apps from which the Google Play page existed and that we gathered during this study and the data we were able to extract from them. The second column gives the country of origin of each app, the third column gives information about the type of developer of each app (e.g., governmental, researcher, company, etc.) and the fourth column gives the target of each app (e.g., citizens,journalists, etc.). Afterwards, the rest of the table is composed of the first category of our taxonomy. We can see that each column of this category is a leaf node of the Information Broadcast branch of the taxonomy (see Fig. 8). A check mark indicates that the corresponding app belongs to this category. Table 3 also represents the list of 78 Covid-related apps, but it shows the mapping between each app and the upstream branch (see Fig. 9). Finally, Table 4 lists all the 78 Covid-related apps with the last part of the leaf node of the upstream branch and the tooling branch (see Fig. 10). 
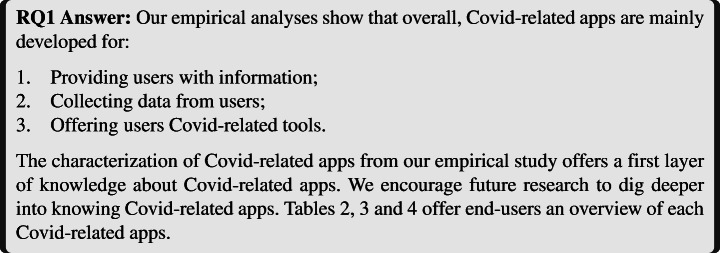


### Do Covid-Related Apps Have Specific Characteristics?

#### Motivation

After categorizing Covid-related apps from their descriptions, in-depth analysis is needed to better understand how they work compared to standard apps. This section aims at comparing Covid-related apps and standard apps from a technical point of view to bring insight into future research. To that end, we extract Android apps-related features (e.g., GUI components, permissions, libraries, etc.). The outcome of this research question will provide the reader with detailed information about Covid-related apps. Indeed, it gives information on whether Covid-related apps are more prone to track and/or display advertisements than standard apps.

#### Strategy

In prior work, Tian et al. (2015) have shown that specific sets of apps can have similar characteristics (e.g., similar permissions, components, size, etc.). In this section, we investigate to what extent 92 apps form one coherent group that is significantly different than other apps.

To that end, for each app, we counted the number of different Android components (i.e., Activities, Broadcast Receivers, Services, and Content Providers), computed the size of the dex file, extracted the permissions needed as well as the libraries used.

#### Comparison dataset

For comparing the characteristics of Covid-related apps with other apps characteristics, we randomly selected 100 apps over 10 different categories of apps from Google Play. Those 1000 (10 x 100) apps are sampled from the same time span (i.e., they are coming from the same initial dataset) to ensure that time is not a factor in potential differences.

Google Play contains dozens of categories, therefore we decided to compare our set of Covid-related apps against apps from the categories that intersect those of our Covid-related apps. Table 5 shows the categories of Covid-related apps we were able to retrieve. Note that we were able to get the category of 87 among our set of 92 Covid-related apps.

**Table 5:** Categories of Covid-related apps and the number of apps in each category

Category	# of apps	Category	# of apps
Communication	2	Entertainment	1
Productivity	1	Medical	23
Health & Fitness	48	Tools	3
Social	3	Lifestyle	4
Shopping	1	Travel & Local	1

#### Android Components

**Fig. 11:**
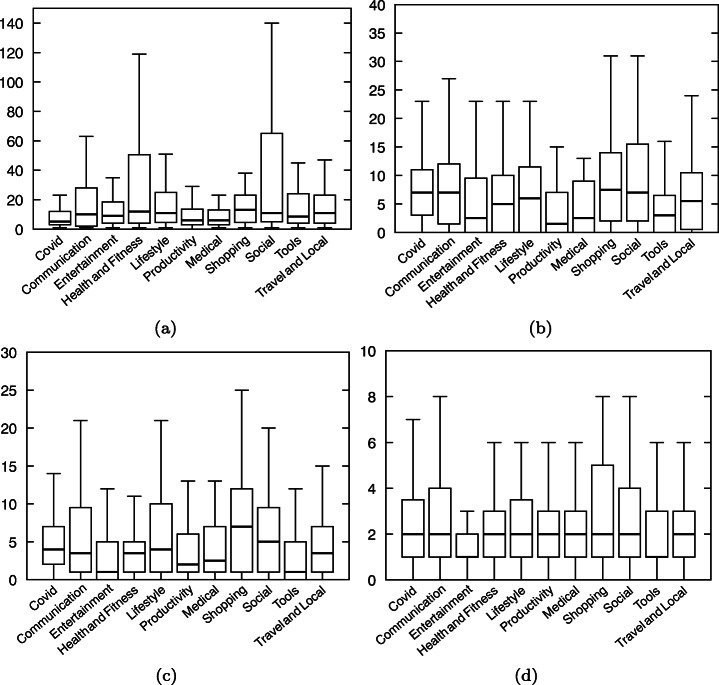
Number of components: Comparison between apps in categories and Covid-related apps

Figure 11 depicts differences between apps in different categories and our set of Covid-related apps regarding the number of components included in the app. We notice that Covid-related apps tend to use fewer Activities than the other apps. This difference is statistically confirmed to be significant by a Mann-Whitney-Wilcoxon (MWW)^12^ test (Mann and Whitney 1947; Wilcoxon 1945)). Regarding Services (used for background tasks), we can see that, apart from the category “Shopping”, Covid-related apps tend to use more service components. Regarding broadcast receivers, however, the difference is less marked, although its statistical significance is confirmed by a MWW test. Finally, the median number of Content providers in Covid-related apps is in most cases equal to the median number of Content providers of apps in different categories (i.e., 2 content providers). An MWW test found no statistically significant difference (except for the *Entertainment* category).

Overall, the differences, which are mostly pronounced for Activities, suggest that Covid-related apps are different from other apps (of the same category) in terms of GUI layout. With less Activities, we can conclude that Covid-related may have less complex GUI than other apps. Services being slightly more used in Covid-related apps, it hints that Covid-related apps are more data-centric than other apps (in the same categories).

#### Dex Files Size

**Fig. 12:**
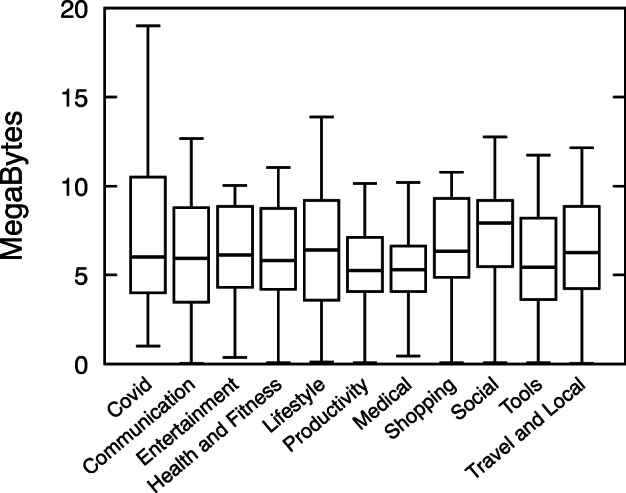
Size of applications: Comparison between apps in categories and Covid-related apps

Figure 12 shows the distributions of the dex sizes of Covid-related apps and the apps from the ten different categories. It shows that the median of Covid-related apps sizes is close to other apps in general. The MWW test confirms no statistically significant difference between the distributions of app size. However, the maximum dex size value is higher than other apps, hinting at more variability in terms of app size amongst Covid-related apps.

#### Permissions

In Table 6, we compare the permissions used by Covid-related apps and the permissions of other apps per app category. To that end, we extracted for all sets of apps the top ten most requested permissions. First, a notable difference is that Covid-related apps tend to use the wake_lock permission more than standard apps. This permission is used for preventing the screen of the device from being turned off, and/or to ensure an app remains active. Such a feature is often used for keeping the phone awake while locating the phone (e.g., for contact tracing). In the same way, access_fine_location and access_coarse_location tend to be used more by Covid-related apps. This is in line with the use of the wake_lock permission to facilitate user location tracking.

**Table 6:** Top ten most requested permissions in Covid-related apps and other apps per category. Percentage indicates the ratio of apps using the permission

Permissions						
	Communication	**%**	Entertainment	**%**	Health & Fitness	**%**
1	INTERNET	100%	INTERNET	98%	INTERNET	99%
2	ACCESS_NETWORK_STATE	97%	ACCESS_NETWORK_STATE	98%	ACCESS_NETWORK_STATE	90%
3	WRITE_EXTERNAL_STORAGE	90%	WRITE_EXTERNAL_STORAGE	74%	WRITE_EXTERNAL_STORAGE	77%
4	READ_EXTERNAL_STORAGE	77%	ACCESS_WIFI_STATE	69%	WAKE_LOCK	67%
5	WAKE_LOCK	76%	READ_EXTERNAL_STORAGE	57%	ACCESS_WIFI_STATE	67%
6	c2dm.permission.RECEIVE	71%	WAKE_LOCK	51%	READ_EXTERNAL_STORAGE	57%
7	ACCESS_WIFI_STATE	62%	RECEIVE_BOOT_COMPLETED	36%	c2dm.permission.RECEIVE	53%
8	VIBRATE	61%	ACCESS_FINE_LOCATION	30%	ACCESS_COARSE_LOCATION	53%
9	CAMERA	61%	c2dm.permission.RECEIVE	29%	ACCESS_FINE_LOCATION	52%
10	RECEIVE_BOOT_COMPLETED	49%	ACCESS_COARSE_LOCATION	29%	RECEIVE_BOOT_COMPLETED	41%
	Productivity	%	Medical	%	Shopping	**%**
1	INTERNET	97%	INTERNET	98%	INTERNET	100%
2	ACCESS_NETWORK_STATE	93%	ACCESS_NETWORK_STATE	92%	ACCESS_NETWORK_STATE	97%
3	WRITE_EXTERNAL_STORAGE	90%	WRITE_EXTERNAL_STORAGE	73%	WRITE_EXTERNAL_STORAGE	84%
4	ACCESS_WIFI_STATE	61%	WAKE_LOCK	55%	WAKE_LOCK	77%
5	READ_EXTERNAL_STORAGE	54%	READ_EXTERNAL_STORAGE	51%	c2dm.permission.RECEIVE	75%
6	WAKE_LOCK	53%	ACCESS_WIFI_STATE	49%	READ_EXTERNAL_STORAGE	68%
7	CAMERA	50%	c2dm.permission.RECEIVE	48%	ACCESS_WIFI_STATE	64%
8	c2dm.permission.RECEIVE	44%	CAMERA	44%	VIBRATE	55%
9	ACCESS_FINE_LOCATION	43%	VIBRATE	41%	RECEIVE_BOOT_COMPLETED	54%
10	ACCESS_COARSE_LOCATION	38%	ACCESS_FINE_LOCATION	39%	CAMERA	54%
	Lifestyle	%	Tools	%	Travel & Local	**%**
1	INTERNET	99%	INTERNET	97%	INTERNET	100%
2	ACCESS_NETWORK_STATE	97%	ACCESS_NETWORK_STATE	91%	ACCESS_NETWORK_STATE	98%
3	WRITE_EXTERNAL_STORAGE	78%	WRITE_EXTERNAL_STORAGE	76%	WRITE_EXTERNAL_STORAGE	84%
4	ACCESS_WIFI_STATE	69%	ACCESS_WIFI_STATE	60%	WAKE_LOCK	76%
5	WAKE_LOCK	67%	READ_EXTERNAL_STORAGE	55%	ACCESS_FINE_LOCATION	70%
6	c2dm.permission.RECEIVE	55%	WAKE_LOCK	48%	ACCESS_WIFI_STATE	68%
7	READ_EXTERNAL_STORAGE	55%	ACCESS_FINE_LOCATION	42%	ACCESS_COARSE_LOCATION	64%
8	ACCESS_FINE_LOCATION	46%	ACCESS_COARSE_LOCATION	40%	c2dm.permission.RECEIVE	63%
9	VIBRATE	44%	VIBRATE	34%	READ_EXTERNAL_STORAGE	60%
10	ACCESS_COARSE_LOCATION	42%	c2dm.permission.RECEIVE	31%	VIBRATE	41%
	COVID	%	Social	%		
1	INTERNET	98.86%	INTERNET	99%		
2	ACCESS_NETWORK_STATE	93.18%	ACCESS_NETWORK_STATE	96%		
3	WAKE_LOCK	78.40%	WRITE_EXTERNAL_STORAGE	87%		
4	c2dm.permission.RECEIVE	73.86%	READ_EXTERNAL_STORAGE	80%		
5	ACCESS_FINE_LOCATION	65.90%	WAKE_LOCK	76%		
6	BIND_GET_INSTALL_REFERRER_SERVICE	59.10%	ACCESS_WIFI_STATE	73%		
7	ACCESS_COARSE_LOCATION	54.64%	c2dm.permission.RECEIVE	69%		
8	RECEIVE_BOOT_COMPLETED	50.00%	ACCESS_COARSE_LOCATION	63%		
9	FOREGROUND_SERVICE	50.00%	ACCESS_FINE_LOCATION	61%		
10	WRITE_EXTERNAL_STORAGE	38.64%	CAMERA	50%		

**Fig. 13:**
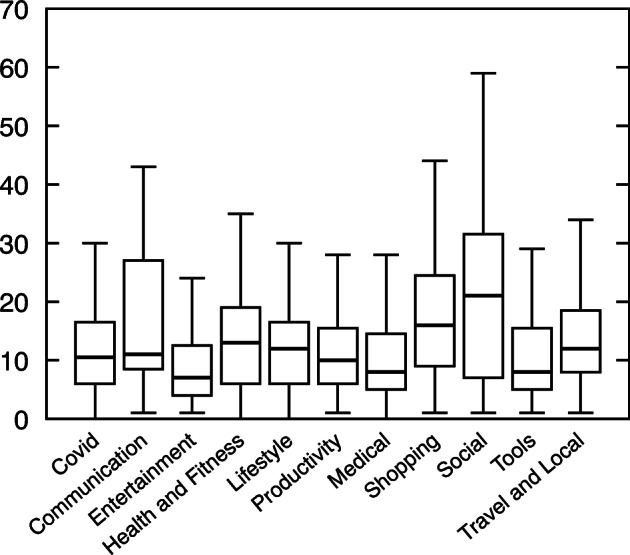
Number of permissions per app

Figure 13 shows the distribution of the number of permissions requested by Covid-related apps and other apps per category. The MWW tests revealed no statistically significant difference between the number of permissions used by Covid-related apps and by other apps

#### Libraries

To compare the patterns of libraries inclusion, we measure the use of libraries by relying on a collection of well-known libraries. More specifically, we re-use two lists of libraries established in prior works (Li et al. 2016, 2019): a list of 1 114 common libraries and a list of 240 advertisement libraries.

Therefore, for Covid-related apps and our dataset of apps by category, we computed the number of apps using at least one common library and one advertisement library.

Table 7 presents our results. First, we notice that almost all the apps (Covid-related and other) use common libraries, which is not surprising since Android software development—just like non-mobile software—heavily relies on reusable libraries and frameworks.

**Table 7:** Number of Covid-related/other apps using libraries. (C: Communication, E: Entertainment, H&F: Health & Fitness, L: Lifestyle, P: Productivity, M: Medical, SP: Shopping, S: Social)

Type of library	COVID	C	E	H&F	L	P	M	SP	S
Common Library	100%	96%	98%	93%	95%	97%	97%	98%	93%
Ad Library	19.6%	93%	98%	87%	92%	83%	86%	93%	86%

However, the difference is significant regarding the advertisement libraries. Indeed, while advertisement libraries are used by more than 80% of other apps, they only appear in less than 20% of Covid-related apps. Furthermore, only 3 out of 240 advertisement libraries are used in Covid-related apps, namely: (1) *com.facebook*, (2) *com.startapp.android* and (3) *com.flurry*. This strongly suggests that the primary goal of Covid-related apps is not to obtain a financial gain from advertisement, in opposition to the vast majority of standard apps. 
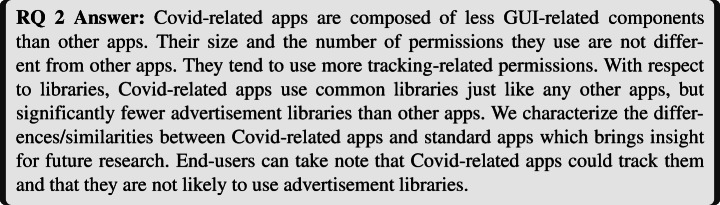


### Are Covid-Related Android Apps More Complex Than Standard Apps?

#### Motivation

In Section 4.1, we have seen that Covid-related apps cover a large variety of categories and target various objectives (e.g., informing users, collecting data from users, etc.). The code complexity that is necessary to achieve these objectives may thus vary substantially. To investigate this aspect, we compute several standard metrics used in the state of the art literature, and further assess the potential differences between Covid-related apps and other apps. Insights from this research question can improve developer’s knowledge and serve as the basis for future empirical research on code quality.

#### Strategy

App complexity is an elusive concept. Yet, in the literature, there are various studies that propose metrics to measure some form of complexity and attempt to show its correlation with app quality and maintainability (Jošt et al. 2013; Gao et al. 2019b). We undertake to investigate our research question based on these common metrics from the literature (Chidamber and Kemerer 1994). We provide in Appendix A the descriptions of the complexity metrics we use.

In this study, the data extracted for computing the complexity metrics are computed at the smali code level. The apps are loaded with Androguard (2020), a static analysis tool for Android apps.

The different metrics attempt to capture the *Lack of Cohesion in Methods* (LCOM), the *Weighted number of Methods per Class* (WMC), the number of methods invoked per class, i.e., the *Response For a Class* (RFC), the *Coupling Between Object classes* (CBO) and the *Number Of Children per class* (NOC).

**Fig. 14:**
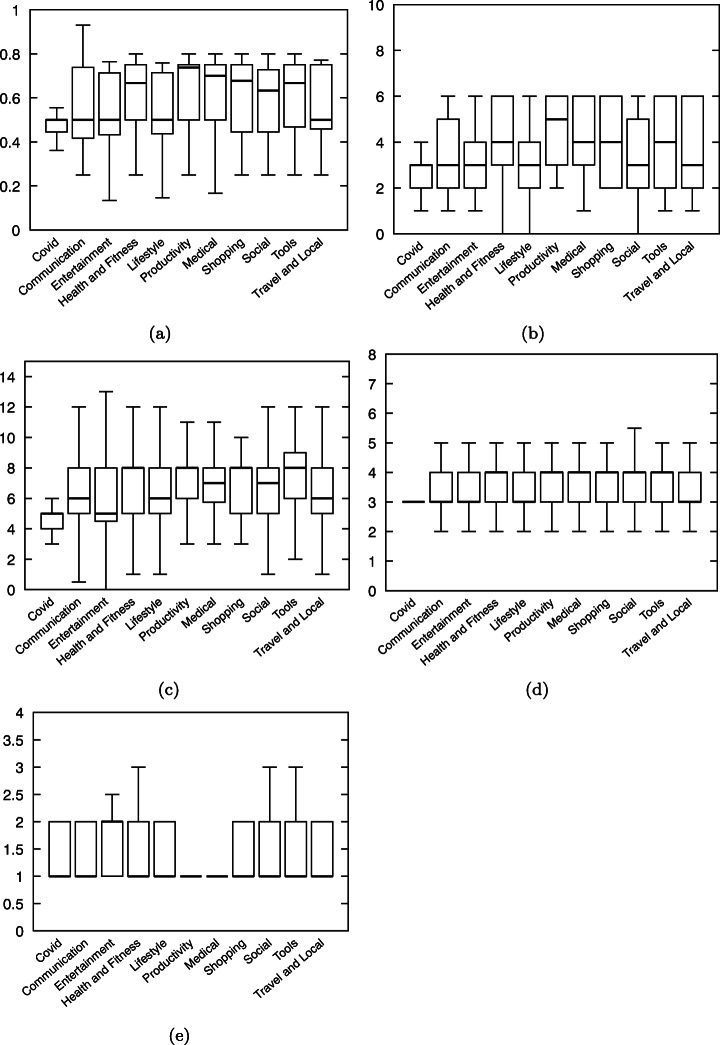
Complexity of Covid-related apps compared to standard apps

Figure 14 presents the distributions of metric values.

NOC appears to present similar distribution across standard and Covid-related apps, confirmed by MWW test. However, MWW test revealed significant differences between the distributions of Covid-related apps and standard apps for the other metrics.^13^

Furthermore, Fig. 14 distinctly shows that Covid-related apps complexity metric medians’ are below standard apps medians which hints at a lower complexity.

We note that obfuscation is a factor that can have an impact on Android apps studies, especially with app complexity computation based on smali code. Our set of Covid-related apps contains 2 apps (2.17%) that contain obfuscated code. For measuring if an app uses obfuscated code, we rely on APKiD.^14^ The obfuscation rate of apps in each category is depicted in Table 8.

**Table 8:** Rate of apps obfuscated by category

Category	Obfuscated apps rate
Covid	2.17%
Communication	18%
Entertainment	20%
Health & Fitness	15%
Lifestyle	16%
Medical	3%
Productivity	5%
Shopping	27%
Social	36%
Tools	34%
Travel & Local	50%

At first sight, we can see in Table 8 that for some categories, there is a high number of apps that contain obfuscated code, which suggests that the metrics computed can be biased by the obfuscation rates.

We therefore conducted the same comparisons, but based on random sets of non-obfuscated apps. The conclusions remain the same.

Overall, these results establish that Covid-related apps are, to some extent, less complex than standard apps. According to (Jošt et al. 2013), this result suggests that Covid-related apps may be more maintainable and of better quality. Additionally, we note that a lower complexity could also indicate that Covid-related apps have on average less functionalities and/or are focused on more specific goals, as was already hinted above in the permission usages comparison. 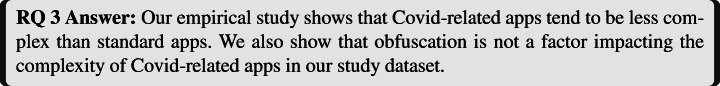


### To What Extent Were Covid-Related Apps Removed From the Official Google Play And Why?

#### Motivation

Developers have to comply with strict Google policies (Google 2020b) before submitting an app to Google Play. The unprecedented crisis of the COVID-19 led Google to release new policies regarding Covid-related apps that would be candidates for Google Play (Google 2020a) where Google performs supplementary checks (e.g., reduce misinformation by favoring official sources). With this research question, we aim to check to what extent Google actually applied strict policies in Google Play. The outcome of this research question will open new research avenues for app policy modeling, and may shed light into Google vetting processes for developers.

#### Strategy

We have seen in Section 4.1 that during our analyses, some Covid-related apps disappeared from the official Google Play in a matter of days.

Therefore, for each app that was initially identified at the beginning of our study, we queried the Google Play market, at the time of writing, to check if the app is still available. Around 15% of Covid-related apps (i.e., 14 apps) have been removed from Google Play.

In comparison, among 1675 standard apps taken randomly from our initial dataset (see Section 2), we found that 277 (i.e. 16.54%) apps were removed from the Google Play market.

The removal rates of both app datasets are close. Actually, we expected a much higher removal rate for Covid-related apps. This relatively low ratio of removal for Covid-related apps could be explained in several ways:

- Google either enforces its policy very quickly or pre-screens (i.e., before it is accepted on the market) each app that is potentially relevant to COVID-19; In that case, apps would either never make it to the market, or would be removed too quickly for AndroZoo crawlers to catch them;
- App developers either rapidly adapted to Google’s policy and/or very few developers proposed apps that conflict with Google’s policy.

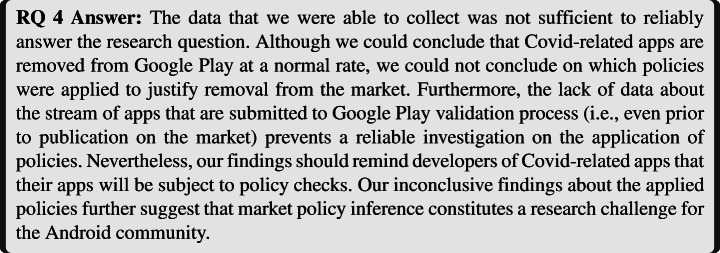


### Who Are Covid-Related Apps’ Developers?

#### Motivation.

In Section 3.2, we have seen that the number of Covid-related apps increased drastically from March 2020. The important information behind this is that many entities quickly responded to the pandemic to provide users with specific Android apps with different purposes (e.g., information, contact tracing, health guide, etc.). However, the nature of the entities was not readily available. In this section, we consider further investigating their type by mining description data and following various links. Typically, we focus on retrieving the origin of those apps to overview what country responded according to the pandemic to provide services to end-users. This information would help to overview which countries quickly reacted to the pandemic by providing end-users with mobile apps services. The outcome of this research question will give the general public a glimpse of the distribution of apps by country. Besides, by exposing the type of developers and the origin of Covid-related apps, we encourage future research into performing additional studies such as code reuse in different apps/countries, plagiarism between apps, as well as correlation between app releases and the number of COVID-19 cases.

#### Strategy.

On Google Play, in each web page of an app,^15^ there is a field *developer* that provides the name of the person or entity (e.g. a software company, a governmental institution, an ONG, etc.) who has released the app. After collecting this information, we detail in Table 2 (column *Developer Type*) the status (or the type) of the entity having released an app. Table 9 presents the number of released Covid-related apps for each type of entities.

**Table 9:** Number of Covid-related apps per entity type

Type of the entity	# of apps	%
Governmental Institution	52	66.67%
Company	17	21.79%
Association	3	3.85%
Independent	2	2.56%
Researchers	2	2.56%
NGO	1	1.28%
Hospital	1	1.28%
Total	78	100%

We can see that most of the app providers are governmental institutions. We indeed find Covid-related apps that are officially promoted by national governments (e.g. Government of Brazil^16^ or Government of France).^17^ We also see apps released by more local governmental bodies (at the state or regional level). We have for instance apps from specific states of the USA (e.g., State of Rhode Island),^18^ or from specific “Switzerland Canton” (e.g. Gesundheitsdepartement des Kantons Basel-Stadt).^19^

About 20% of the Covid-related apps (17 apps) are provided by companies. In order to understand why these apps have not been removed by Google, we further check the description of these apps and the descriptions of the companies. We found that:

- Even if the developer is identified as a company, two apps have been developed on behalf of official bodies (*Care19*^20^ is the official COVID-19 app for the states of South Dakota and North Dakota, *COVID AP-HM*^21^ is an app developed for a hospital);
- Seven apps are either endorsed by a ministry,^22^ or working in close collaboration with medical/health actors,^23^ or working in collaboration with renowned universities.^24^
- Two apps are actually online shopping apps.^25^
- One app is not on the market anymore.^26^
- Finally, five apps related to social distancing,^27^ or health,^28^ or Covid-related news,^29^ have been released by companies without any explicit link to official organizations. We remind that the official Google COVID-19 policy (Google 2020a) is that Covid-related apps with no explicit links with governmental bodies or health organizations cannot provide “health claims”. We further check these 5 apps, and we confirm that they comply with the Google COVID-19 policy.

For the remaining nine Covid-related apps, we noticed that 3 apps have been provided by associations. More specifically, the *DiagnoseMe*^30^ app has been released by the Faso Civic association from Burkina Faso, the *Self Shield App*^31^ by the Commonwealth Medical Association (through the Commonwealth Centre for Digital Health organization) and the *COVID Safe Paths*^32^ app by a non-profit organization related to MIT. We also noticed that two apps have been developed by independent developers, and two other apps have been provided by researchers. One by a group of researchers from German Universities,^33^ one by researchers from the Aga Khan University in Pakistan.^34^ Finally, one app has been provided by an NGO (i.e., the Austria Red Cross), and one by a hospital (actually a group of hospitals in Paris, France).

We note that among all the Covid-related apps, 71% of them have been released by entities having multiple Android apps on Google Play.

**Fig. 15:**
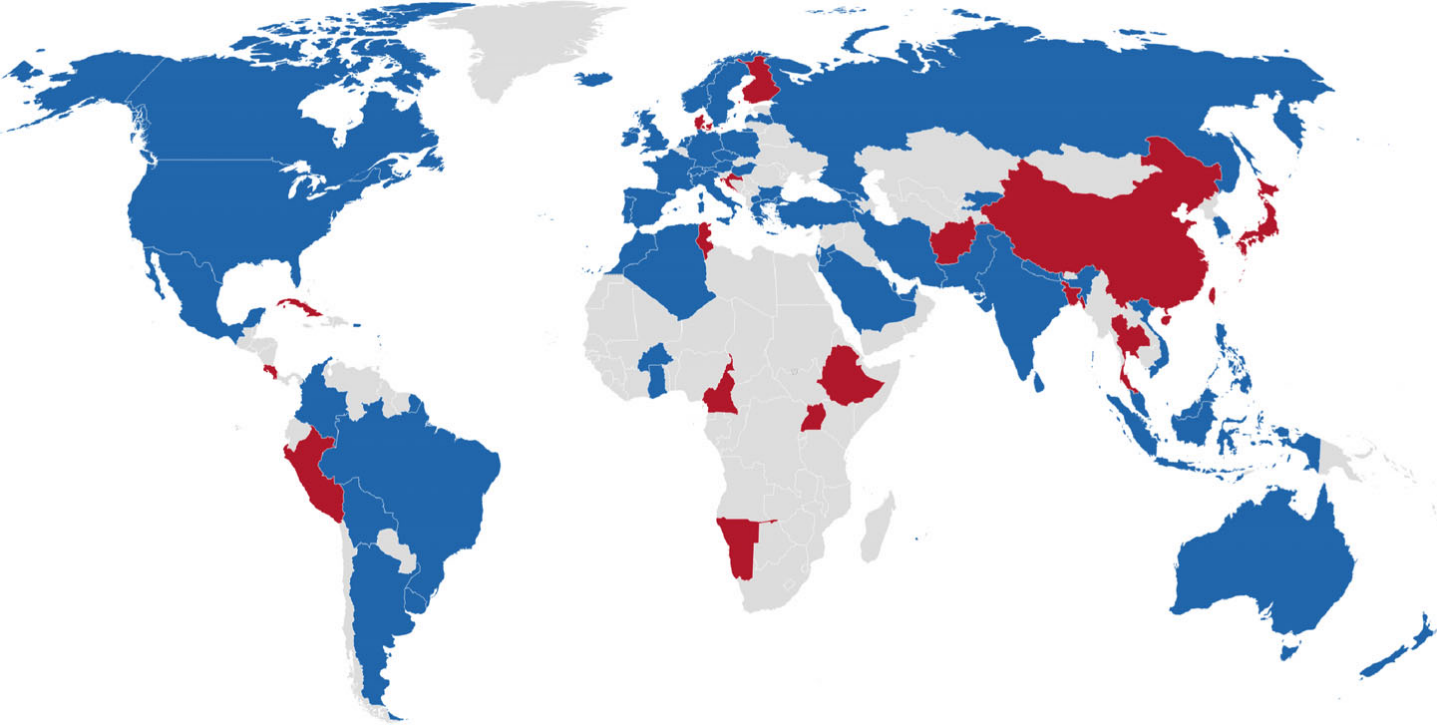
Countries of origin of Covid-related apps (Blue: Apps available, Red: Apps not available)

Finally, we represent in the map of Fig. 15 the geographical distribution of the apps over the world. We can see that Covid-related apps are provided world-wide (maybe less present in Africa). The countries in blue are the ones listed in Table 2. Note that we also identified 16 other apps from 16 countries that we were unable to obtain; These countries are represented in red. 
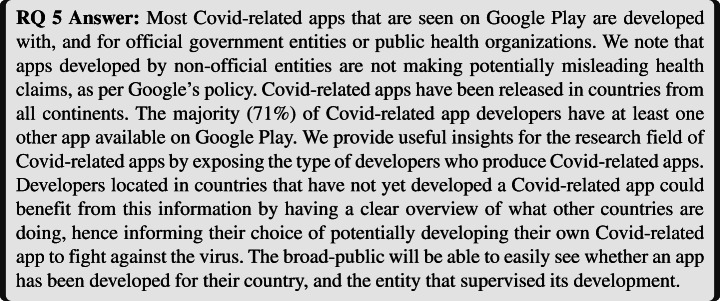

### Do Covid-Related Apps Have Security Issues?

#### Motivation

Security and privacy are critical concerns regarding mobile apps. In this section, we assess several aspects of Covid-related apps security. The outcome of this research question will provide the general public with a summary of some potential security problems found in Covid-related apps, which may help them adjust their level of trust in such apps. Similarly, we highlight potential issues that developers should consider in terms of security of Covid-related apps. Researchers may also use this information to adopt further investigation topics related to Covid-related apps security, e.g., on API usage patterns, evolution of security and privacy in the lineage of apps, etc.

#### Strategy

In contrast to a recent work (He et al. 2020), which focused on dissecting Covid-related malware, our aim in this work is not to perform an extensive security analysis of these apps. Nevertheless, we propose to leverage four practical security and privacy scanners on our set of 92 Covid-related apps in order to systematically evaluate four S&P aspects: (1) the presence of privacy leaks; (2) the number of apps flagged by VirusTotal; (3) the misuse of crypto-APIs; (4) the matching between descriptions and behavior.

**[Privacy leaks]** As we have seen in Section 4.1, most of the Covid-related apps are made for collecting personal and sensitive data, e.g., health data and/or the location of users. Therefore, the security and privacy aspects of these apps are crucial, and many people started to share concerns related to this topic (Page 2020; Parliament 2020; Stolton 2020). In order to assess the privacy of Covid-related apps, we applied the state-of-the-art data leak detector FlowDroid-IccTA (Arzt et al. 2014; Li et al. 2015). Through static analysis, this tool is able to detect sensitive data leaks intra-component (e.g., inside an Activity) or inter-component (e.g., across Activities). Note that we used the default sources and sinks provided with the tool.

FlowDroid-IccTA was able to detect 24 intra-component data leaks in 2 different apps and found no inter-component leak for the list of the 24 leaks. The app *SODI*^35^ contained only 1 potential leak, whereas the app *Coronavirus - SUS*^36^ contained 23 potential leaks. Given that static analysis tools are subject to false-positives, we undertake to manually analyze every detected leak.

We compiled the list of the 24 leaks in Table 10. In the second column we expose the source of the potential leak, i.e., the sensitive information which is the first chain link. The third column lists the sinks associated with the sources, i.e., the method that is responsible for leaking the sensitive information.

**Table 10:** List of the leaks detected by Flowdroid-IccTA. Note that there can be multiple leaks for each couple of source/sink

	Sources	Sinks
	com.bloomreality.sodi	
1	android.content.Context	android.content.Intent.registerReceiver
	br.gov.datasus.guardioes	
1	android.content.Intent.getIntent	android.util.Log.w
2	android.os.Bundle	android.util.Log.w
3	android.content.Context	android.util.Log.d
4	android.content.Context	android.util.Log.d
5	java.net.URL.openConnection	android.util.Log.e
6	java.net.URL.openConnection	android.util.Log.e
7	android.location.Location.getLongitude	android.util.Log.d
8	org.apache.cordova.CordovaActivity.getIntent	android.util.Log.d
9	android.location.Location.getLongitude	android.util.Log.d
10	java.net.URL.openConnection	android.util.Log.d
11	java.net.URL.openConnection	android.util.Log.d
12	android.location.Location.getLatitude	android.util.Log.d
13	android.os.Bundle	android.util.Log.d
14	java.net.URL.openConnection	android.util.Log.d
15	android.location.Location.getLatitude	android.util.Log.d
16	android.content.Intent	android.util.Log.d
17	android.content.Intent	android.util.Log.d
18	com.google.firebase.messaging.RemoteMessage	android.util.Log.d
19	android.content.Intent	android.util.Log.d
20	android.content.Intent	android.util.Log.d
21	org.apache.cordova.CordovaActivity.getIntent	android.util.Log.d
22	android.os.Bundle	android.util.Log.d
23	java.net.URL.openConnection	android.util.Log.d

*SODI* an app promoting social-distancing. The app is not originating from a government. Our manual analysis concluded, however, that the reported leak is a false-positive alarm and does not constitute a real data leak.

Regarding *Coronavirus - SUS*, which is an official app of the government of Brazil, FlowDroid-IccTA flagged 24 potential sensitive leaks (i.e., there is a path between a source (that can access a sensitive data) to a sink (e.g. sendTextMessage)). We notice that four of these leaks allow the app to get the longitude and/or latitude (the sources) of the app to log it internally (the sink). However, this does not necessarily constitute a malicious behavior.

**AntiVirus detection**: For each of the Covid-related apps, we have collected the detection reports from over 60 AntiVirus products, thanks to the VirusTotal API.^37^ None of Covid-related apps is flagged by any of the 60 anti-virus software available in VirusTotal at the time of writing.
**Crypto-API misuses**: Finally, we leverage the state-of-the-art static-analyzer CogniCrypt (Krüger et al. 2017) through its headless implementation CryptoAnalysis (CryptoAnalysis 2020) for detecting cryptographic API misuses in Java programs. Such misuses could indeed indicate security issues. We found that 81 apps among our set of 92 Covid-related apps use JCA^38^ APIs. However, CogniCrypt did not report any cryptographic misuse.
  In contrast, Gao et al. (2019a) have shown that in a dataset of more than 598000 apks, 96% of apks using JCA exhibit dangerous misuses of cryptographic APIs. With 0%, Covid-related apps seem to be totally exempt from such misuses.
**Description/Behavior matching**: Covid-related apps may propose functionalities that are sensitive due to the security and privacy concerns that they can raise (e.g., the Indian app Aarogya Setu (Clarance 2020) was found to share users’ private information to third parties). The apps we study have been released on Google Play, therefore users can only rely on the description provided by developers.

However, it has been shown by Gorla et al. (2014) that apps’ behavior does not always match the apps’ description. For this reason, we replicated the CHABADA approach(Gorla et al. 2014) to check to what extent Covid-related apps’ descriptions match their behavior (approximated by API usages). CHABADA unfolds as follows:

1. Preprocessing descriptions with NLP techniques: tokenization, stop word removal, stemming
2. Extracting topics with Latent Dirichlet Allocation (Blei et al. 2003)
3. Clustering apps based on topics with K-means (MacQueen 1967)
4. Identifying, in each cluster, the apps that have outlier API usages. This outlier identification is performed via One-Class Support Vector Machine learning (Schölkopf et al. 2001)

In Section 4.1, we have seen that we were able to retrieve the descriptions of 78 apps. We applied our implementation of CHABADA to these 78 apps. The clusters generated by CHABADA can be seen in Table 11. Five clusters have been generated. We have named these clusters by considering the three most used words per cluster. We can see that the first cluster (i.e., Spread tracking) contains 30 apps, whereas other clusters are smaller and are all roughly the same size (i.e., between 10 and 14 apps per cluster). Do note that the clusters, which are independently built using the CHABADA approach, can each be associated to a category of our taxonomy (defined in Section 4.1).

**Table 11:** Clusters of apps generated by our implementation of CHABADA

id	Cluster	# of apps	# outliers
0	Spread tracking	30	3
1	Sharing health information	12	2
2	Sharing general information	10	4
3	Data collection	12	4
4	COVID-19 self-diagnosis	14	3

After clustering the apps based on their description, CHABADA searches for outliers in each cluster based on the APIs usage. Table 11 shows the number of outliers detected per cluster. The three outliers in “Spread tracking” have been detected because, contrary to other apps in the cluster, they use Android vibration API and Android MediaPlayer API. Similarly, in the cluster “Sharing health information”, the two outliers use Bluetooth APIs, which is not the case for other apps in the cluster. Regarding the cluster “Sharing general information”, the outliers use the SmsManager API, the TelephonyManager API, and the SpeechRecognizer API. In the “Data collection” cluster, the use of the Bluetooth API is common: outliers do not use this API. Finally, in the “COVID-19 self-diagnosis” cluster, the outliers use the MediaPlayer API, which is not used by the rest of the cluster apps.

CHABADA allowed us to identify several Covid-related apps that deviate from the expected behavior given their description. Although the outliers do not necessarily present a danger for end-users, because, in general, they only deviate with respect to non-sensitive APIs (e.g., MediaPlayer, Vibrator, etc.), our empirical results show that descriptions do not always reliably approximate the expected app behavior. 
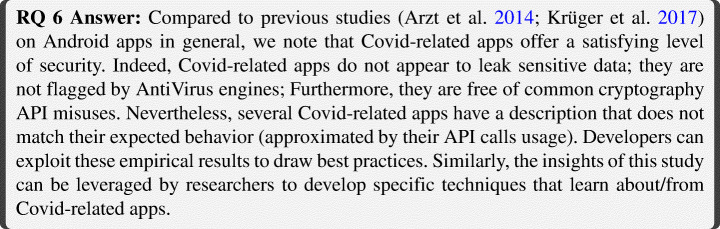


## Threat to Validity

Our study bears a number of threats to validity related to the selection of apps, external factors that may impact our conclusions, and the limitations of the leveraged tools.

### Covid-Related Apps Retrieval

#### We may have missed some Covid-related apps.

Our approach relies on simple heuristics to gather applications from AndroZoo, which helped to identify 44 unique apps. Therefore, we cannot guarantee that our app collection of Covid-related apps is exhaustive. However, we leveraged AndroZoo which is the largest and continuously-updated repository of Android applications available to the research community, which we further manually supplemented with other sources. Overall, it is unlikely that we missed a number of Covid-related apps that is large enough to significantly invalidate the conclusions presented in this paper. Actually, as we have revealed, we were able to catch in time some apps that were later removed from the market.

#### The enforcement of Google Policy on market apps may have biased our study on Covid-related apps

Our experiments were conducted until early June 2020, and we know that Google has been enforcing its policy regarding Covid-related apps since at least early April (Google 2020a). Given our vantage point without insider knowledge, our observations are limited to apps that were actually released on Google Play, and our study is blind to apps that were never let in Google Play. There is thus a possibility that some of our findings are *consequences* of Google’s policy, and not a characterization of apps that were meant to be on Google Play. Nonetheless, our study reflects closely the landscape of apps that were made available to users.

On the other hand, we note that the removal of apps from the market may have been performed by either market maintainers or by developers themselves. Future studies may attempt to investigate closely the reasons of app removal.

#### Apk date is unreliable

In our collected dataset, the earliest date of appearance of Covid-related apps goes back to September 2017, but we relied on the earliest date between the AndroZoo added date, the first submission date to VirusTotal, and several other websites (included last update date in Google Play as discussed in Section 3.2). Since AndroZoo does not provide the release date of an app (which is different from the added date), it could be the case that the apps existed before. Unfortunately, we cannot rely on the dates of the files constituting the apps since they can easily be modified (Li et al. 2018). Fortunately, Google Play mirrors exist (e.g., appbrain.com) and can keep track of different versions of Android apps, even the first release date. We heavily relied on those dates which mitigates the threats to validity.

### Reliability of the App Description

As discussed in Section 3.1, some parts of our study on Covid-related apps require manual analysis, e.g., to inspect app’s descriptions or app’s content, or to check the output of the automated analysis tools. While we followed a consistent process by carefully verifying what was under study and by replicating this process multiple times, the conclusions are subject to human subjectivity.

### Reliability of the Manual Analyses

As discussed in Section 3.3.3, some parts of our study on Covid-related apps require manual analysis, e.g., to inspect app’s descriptions or app’s content, or to check the output of the automated analysis tools. While we followed a consistent process by carefully verifying what was under study and by replicating this process multiple times, the conclusions are subject to human subjectivity. Therefore, the results presented (e.g. the taxonomy) may not be as representative as they should be. Nevertheless, we strongly believe that, from the process we followed, it is unlikely that the results we collected are not far from the truth.

### Tools Limitations

We leveraged several security scanners to evaluate different security characteristics of apps, and hence inherit their limitations. We cannot guarantee that these tools yielded accurate analyses. We mitigated the threats by first ensuring the selection of state of the art tools that are commonly used in the literature, and second by ensuring that we do not overclaim based on their results.

## Related Work

Several prior works have conducted empirical studies on large sets of Android applications collected from app markets. Viennot et al. (2014) collected more than a million apps from Google Play and uncovered several interesting patterns in Android apps and the way they are developed. Also in 2014, Gorla et al. (2014) collected apps and their associated descriptions and automatically verified whether the descriptions actually matched the app behaviors while Qu et al. (2014) checked the descriptions against the permission usages. Other works focused on financial apps (Taylor and Martinovic 2017), on app maintenance and prices (Carbunar and Potharaju 2015), on malware (Zhou and Jiang 2012), or on the quality of apps descriptions (Jiang et al. 2014).

To the best of our knowledge, the academic literature has not yet reported on a systematic study on Covid-related Android apps. He et al. (2020) however have recently discussed the case of coronavirus-themed malicious apps. In this paper, the authors analyzed 277 malicious applications related to COVID-19 among an initial dataset of 2016 applications. In our paper, we did not identify any malicious Covid-related apps which seems to be in contradiction with He et al. (2020) who collected 277 malicious Covid-related apps. However, this discrepancy could be explained by at least two reasons:

- We used a more selective filtering process to collect Covid-related apps, notably because we realized that the keywords we initially used (and that He et al. (2020) also used) were too broad, i.e., they catch many apps not related to COVID-19;
- Among their initial dataset of 2016 apps, only 6 are coming from Google Play. Unfortunately, the paper does not precise whether the 6 apps from Google Play are part of their 277 identified malicious apps. Overall, their results show that the vast majority (and probably all, as per our results) of malicious Covid-related apps are coming from application sources that are outside Google Play.

Besides, note that in He et al. (2020) we do not have the information whether they considered different versions of a single app or not. Considering multiple versions could drastically lower the number of apps in their dataset. Their study further shows that there is a correlation between the number of COVID-19 cases in the world and the number of malicious Covid-related apps and that malicious developers did not repackage existing Covid-related apps for releasing malicious apps, contrary to a common malware practice (Zhou and Jiang 2012). He et al. (2020) uncovered 34 different malware families used in malicious Covid-related apps. Their results further suggest that malware developers do not target specific users but target a wide range of countries.

Since the emergence of the COVID-19 topic in the media, researchers are investigating its effect, not only on the medical front but also on our daily life. Related to the security perspective of mobile applications users, several security researchers and analysts publicly disclosed their findings, usually in blog posts or press articles, about the activity of malware developers trying to take advantage of the COVID-19 crisis (Doffman 2020; Buguroo 2020; Saleh 2020; Arsene 2020).

Similarly, from a privacy perspective, a common functionality of Covid-related apps for fighting against the spread of COVID-19 is contact tracing. Tracing however carries several concerns with respect to user privacy. Indeed, Baumgärtner et al. (2020) show that although developers claim to respect privacy, it is possible to de-anonymize information about infected persons that are traced and even sabotage the tracing effort by injecting fake contacts.

## Conclusion

In this paper, we provide a first systematic study of Covid-related Android applications. We collected from different channels 92 apps that are manually vetted as relevant. Then, based on the apps’ described goals, our study yields a taxonomy of Covid-related Android apps as a contribution to the literature. Our empirical findings reveal that Covid-related apps have mainly three purposes: (1) inform users, (2) collect data, and (3) provide tooling capabilities for users. After exploring the inner characteristics (e.g., libraries, permissions, size) and the results of security and privacy scanners, we provide first insights into the nature of Covid-related apps. Overall, our empirical study constitutes a first milestone towards understanding who, what, and how Covid-related apps are built. We expect future work in the community to go in-depth into each of the dimensions that we have explored.

All artifacts are made available online at:

https://github.com/JordanSamhi/APKCOVID

## Appendix A: Description of complexity metrics

Below we present five complexity metrics initially described by Chidamber and Kemerer (1994).

### A.1 Weighted Methods per Class (WMC)

This metric represents the complexity of individual classes by counting the number of methods per class. Its purpose is to estimate how challenging the development and maintenance of a class are. Also, as the value for a given class grows, the impact on a class inheriting it will be greater as they will inherit all the methods.

### A.2 Number Of Children (NOC)

This metric represents the number of immediate sub-classes of a class in the class hierarchy. It measures how many sub-classes will inherit the features (fields, methods, etc.) of the parent class. The greater the number of children, the greater the reuse of properties, meaning that it may require more testing.

### A.3 Lack of Cohesion in Methods (LCOM)

This metric represents, for each data field in a class, the average percentage of the methods using that field. It measures the degree of cohesion between methods of a class and data fields of the given class. The lower the cohesion, the greater the complexity as it increases the probability of errors during the development process.

### A.4 Coupling Between Object classes (CBO)

This metric represents the dependency that a class has on other classes. It is computed by counting the number of classes used in a given class. The greater the value, the greater the complexity since it implies high dependency, hence more testing, and less reusability,

### A.5 Response For a Class (RFC)

This metric represents the number of unique method calls within a given class. It is computed by counting all the method invocations in a class only once. The greater this metric, the greater the complexity since it implies a greater level of understanding for the tester for debugging and/or testing purposes.

## Footnotes

1 https://en.wikipedia.org/wiki/COVID-19_apps

2 https://coronalabs.com/

3 https://koodous.com

4 https://apkcombo.com

5 https://apkpure.com

6 https://appbrain.com

7 https://github.com/androguard/androguard

8 The description of an app can be retrieved from the web page of the app on Google Play. This web page can be accessed with the following link: https://play.google.com/store/apps/details?id=PACKAGENAME, where PACKAGENAME is the package name of the app (e.g. *fr.gouv.android.stopcovid*).

9 https://github.com/skylot/jadx

10 package name: *app.ceylon.selftrackingapp*

11 package name: *bf.diagnoseme.fasocivic*

12 All MWW tests are done with significance level set at 0.05

13 WMC ($\bar {p} = {3.48e-05}$), RFC ($\bar {p} = {5.93e-06}$), CBO ($\bar {p} = {1.1e-04}$) and LCOM ($\bar {p} = {1.5e-02}$)

14 https://github.com/rednaga/APKiD

15 https://play.google.com/store/apps/details?id=PACKAGENAME, where PACKAGENAME is the package name of the app.

16 br.gov.datasus.guardioes

17 fr.gouv.android.stopcovid

18 com.ri.crushcovid

19 ch.covid19bs.app.PMSMobile

20 com.proudcrowd.care

21 com.ambulis.aphm.covid

22 covid19care.virus.coronavirus.corona.sick.marcom.health.pakistan

23 com.docandu.checker, it.adl.aslroma3.covid19.app, it.adilife.covid19.app, com.maithu.transplantbuddy.covid19, de.bssd.covid19

24 com.joinzoe.covid_zoe

25 com.coronavirus.facemask and com.krrsoftwaresolutions11.Facemasks

26 com.dawsoftware.contacttracker

27 world.coalition.app and com.bloomreality.sodi

28 de.kreativzirkel.coronika and it.softmining.projects.covid19.savelifestyle

29 com.osapps.covid19

30 bf.diagnoseme.fasocivic

31 org.sshield.selfshield

32 org.pathcheck.covidsafepaths

33 com.coronacheck.haugxhaug.testyourcorona

34 com.edu.aku.akuhccheck

35 com.bloomreality.sodi

36 br.gov.datasus.guardioes

37 https://www.virustotal.com

38 Java Cryptography Architecture, the most popular library of java Crypto-APIs
